# Network pharmacology combined with metabolomics to explore the mechanism for *Lonicerae Japonicae* flos against respiratory syncytial virus

**DOI:** 10.1186/s12906-023-04286-0

**Published:** 2023-12-12

**Authors:** Jie Ding, Jing Li, Zhe Zhang, Yaxuan Du, Yuhong Liu, Ping Wang, Haitao Du

**Affiliations:** 1 College of Pharmacy, Shandong University of Traditional Chinese Medicine, Jinan, 250355 China; 2 School of Chinese Materia Medica, Shenyang Pharmaceutical University, Shenyang, 117004 China; 3 Shandong Academy of Chinese Medicine, Jinan, 250014 China

**Keywords:** *Lonicerae japonicae* flos, Respiratory syncytial virus, Active ingredient, Network pharmacology, Metabolomics

## Abstract

**Background:**

Respiratory Syncytial Virus (RSV) stands out as a primary contributor to lower respiratory tract infections and hospitalizations, particularly in infants. *Lonicerae japonicae* flos (LJF), a traditional Chinese medicine renowned for its efficacy against various viral infections, including RSV, has been widely employed. Despite its common use, the precise therapeutic mechanism of LJF against RSV remains elusive. This study aimed to investigate the underlying mechanism of LJF against RSV through network pharmacology and metabolomics.

**Methods:**

In this study, based on network pharmacology, potential targets related to LJF and RSV were obtained from PubChem and Swiss Target Prediction. The core targets and pathways were established and verified by enrichment analysis and molecular docking. The anti-RSV efficacy of LJF was determined by in vitro experiments. Additionally, metabolomics analysis was integrated, allowing for the identification of differential metabolites and their correlation with targets following LJF treatment in the context of RSV infection.

**Results:**

A total of 23 active ingredients and 780 targets were obtained, of which 102 targets were associated with LJF anti-RSV. The construction of the corresponding Protein–Protein Interaction (PPI) network unveiled potential core targets, including STAT3, TNF, and AKT1. Gene Ontology (GO) and Kyoto Encyclopedia of Genes and Genomes (KEGG) analysis revealed that LJF's anti-RSV effects primarily involve key pathways such as the PI3K-Akt signaling pathway, EGFR tyrosine kinase inhibitor resistance, and FoxO signaling pathway. Molecular docking showed that ZINC03978781, 4,5'-Retro-.beta.,.beta.-Carotene -3,3'-dione, 4',5'-didehydro and 7-epi-Vogeloside had better binding ability. The cellular assay showed that the therapeutic index of LJF against RSV was 4.79. Furthermore, 18 metabolites were screened as potential biomarkers of LJF against RSV, and these metabolites were mainly involved in the pathways of purine metabolism, linoleic acid metabolism, alpha-linolenic acid metabolism, and other related pathways.

**Conclusions:**

The intergration of network pharmacology and metabolomics can clarify the active targets and related pathways of LJF against RSV, which could provide a valuable reference for further research and clinical application of LJF.

**Supplementary Information:**

The online version contains supplementary material available at 10.1186/s12906-023-04286-0.

## Introduction

Respiratory Syncytial Virus (RSV) is a highly contagious respiratory virus that is the major cause of lower respiratory tract infections in infants and children [1]. Symptoms of RSV infection in children may include fever, runny nose, and cough, and can eventually lead to bronchitis or pneumonia, which can be severe and fatal [2, 3]. In the post-COVID period, RSV infection rates in children are also on the rise, which could be attributed to the prolonged period of minimal RSV exposure, also known as RSV immunity debt [4]. Although the pathological mechanisms of RSV have been studied to some extent, there is still no efficient vaccine or specific antiviral drug [5]. Its inter-regulatory role with host cells, especially at the metabolic level, remains unclear.

*Lonicerae Japonicae* flos (LJF) refers to the dried flower buds or first bloomed flowers of *Lonicera japonica* Thunb. Originating from the ancient Chinese medical book called *Mingyi Bie Lu (Records of Famous Physicians)*, LJF is classified as a top-grade traditional Chinese medicine, known for its efficacy in clearing heat, detoxifying, dispersing wind, and alleviating heat. Moreover, LJF plays a significant role in various health-related activities, including antiviral, anti-inflammatory, antibacterial, immunomodulatory, and antioxidant effects [6]. At present, more than 600 components have been isolated from LJF, including volatile oils, organic acids, flavonoids, polysaccharides, cyclic enol ether terpenoids, saponins, and trace elements [7]. LJF belongs to the category of medicinals with both therapeutic and dietary properties, characterized by low toxicity and minimal side effects. Therefore, it is more worthy of promotion and utilization. In traditional daily life, LJF is often employed in tea consumption to exert its function of clearing heat and detoxification [6]. In recent years, the anti-RSV, H1N1, and severe acute respiratory syndrome coronavirus (SARS-CoV-2) activities of LJF and its extracted components have been gradually discovered [8–10]. During the COVID-19 pandemic, LJF has been commonly utilized in China as a crucial medicine, incorporated into traditional Chinese medicine formulations such as Lianhua Qingwen Capsules and Jinhua Qinggan Granules, exhibiting antiviral properties [11]. With the progression of research, an increasing number of LJF extracts have been demonstrated to possess anti-RSV effects. Ling et al. developed a quantitative analysis method based on Ultra-High-Performance Liquid Chromatography coupled with Triple Quadrupole Mass Spectrometry (UHPLC-QQQ MS). Through the integration of Spearman and Grey analyses, isochlorogenic acid B, isochlorogenic acid C, and secoxyloganin are postulated to be the primary effective constituents of LJF in the treatment of RSV [12]. Li et al. observed that LJF exerts its antiviral effects against respiratory syncytial virus type 3 primarily through direct inactivation, inhibition of virus adsorption, and suppression of biosynthesis in vitro [13].

In recent years, developments in metabolomics and network pharmacology have provided new perspectives and approaches to the study of viral infectivity. Metabolomics is a science that studies the composition and dynamics of all metabolites within a biological system and can provide comprehensive information about a biological system under specific conditions [14]. Since viruses need to rely on host metabolic processes to complete their replication process, they are an excellent choice to study by metabolomics techniques [15]. Network pharmacology, as a new approach to studying the mechanism of action and efficacy of drugs, can reveal the multi-target actions and systemic effects of drugs by constructing drug-target-disease network models [16–18]. This study aimed to explore the mechanism of anti-RSV in LJF by metabolomics and network pharmacology.

## Materials and methods

### Reagents and materials

*Lonicerae Japonicae* flos was purchased from Jianlian Traditional Chinese Medicine Shop (Bozhou Yonggang Drinking Tablet Factory Co., Ltd.) and authenticated by researcher Huibin Lin of Shandong Academy of Traditional Chinese Medicine.

The human laryngeal cancer cell line (Hep-2) was provided by the Institute of Basic Medical Sciences, Shandong Academy of Medical Sciences, Shandong Province, China. BCA protein detection kit (Shanghai Beibo Biotechnology Co., Ltd., batch no. BB20081), methanol (Fisher Chemical, batch no. A4524), and ethyl ether (Fisher Chemical, batch no. A998-4) were purchased from Fisher Chemical Company. The reagents were purchased from Fisher Chemical Company.

### LJF compound-related target screening

The chemical composition of LJF was obtained through The Traditional Chinese Medicine Systems Pharmacology Database (TCMSP, https://tcmsp-e.com/tcmsp.php). According to "ADME screening" under "TCMSP User Guide", set the screening parameters to oral bioavailability (OB) ≥ 30% and drug-likeness (DL) ≥ 0.18. The canonical SMILES structure format of the identified compounds was obtained from the PubChem database (https://pubchem.ncbi.nlm.nih.gov/) and uploaded to the Swiss Target Prediction platform (http://www.swisstargetprediction.ch/) to predict the compound targets. The species restriction was set to "Homo sapiens" and "probability > 0".

### RSV-related target screening

Through the study of GeneCards (https://www.genecards.org/), DisGeNET (https://www.disgenet.org/home/), and OMIM (https://omim.org/) databases, the keyword "Respiratory Syncytial Virus" was used as the keyword for the key targets of RSV infection were searched and analyzed, and summarized with the RSV target database constructed by the group in the previous stage [19], and then false-positive and duplicated targets were deleted and integrated to finally obtain the RSV pneumonia target database. The intersection of compound targets with RSV pneumonia targets was selected as a potential therapeutic target for LJF against RSV.

### Protein–Protein Interaction (PPI) network analysis

The STRING 11.0 database (https://cn.string-db.org/) was used to construct and analyze PPI networks of potential therapeutic targets. Species were restricted to "Homo sapiens" with a minimum interaction score greater than 0.4 (highest confidence level). The PPI network was plotted and visualized for analysis in Cytoscape 3.7.2.

### Enrichment analysis

The DAVID 6.8 database (https://david.ncifcrf.gov/) was applied to conduct the Gene Ontology (GO) and Kyoto Encyclopedia of Genes and Genomes (KEGG) pathway enrichment analysis. The filtering condition was set at *p* < 0.05 and the results were visualized and analyzed by bioinformatics platform (https://www.bioinformatics.com.cn/). In GO enrichment analysis, it includes cell component (CC), biological process (BP), and molecular function (MF) three aspects.

### Molecular docking

The top three proteins in the PPI network were selected for search. The relevant screening criteria are as follows: (1) The structure can find reports related to molecular docking; (2) The structure is as clear as possible (Resolution < 3Å); (3) The molecular system preferably contains co-crystallized ligands. The crystal structures of the core targets STAT3 (PDB ID: 5AX3), TNF (PDB ID: 4TSV), and AKT1 (PDB ID: 1UNQ) were downloaded from the RCSB PDB database [20–22]. Secondly, the two-dimensional structures of the LJF components were obtained from the PubChem database (https://pubchem.ncbi.nlm.nih.gov/), and ligand molecules were prepared. AutoDock Vina (http://vina.scripps.edu/) was used for molecular docking to find the optimal conformations and calculate their binding affinities. The docking results between the active components and the main protein targets were visualized using the PLIP platform (https://plip-tool.biotec.tu-dresden.de/).

### Extraction of LJF aqueous solution

LJF (50 g) was extracted with the reflux extraction method as follows. 50 g of medicinal materials were soaked in distilled water (1: 10, w/v) for 1 h, and then extracted twice with 85 ℃ hot water for 45 min each time. The filtrates were combined, concentrated to contain 1 g/mL of raw herb, sterilized, and stored at -20 ℃.

### Inhibitory effect of LJF on RSV

Hep-2 cells were inoculated into culture dishes at a density of 1 × 10^5^ cells/L, then LJF was added at the maximum nontoxic concentration and incubated for 24 h. Each dish was inoculated with 1 mL of 100 TCID50 RSV and the cells were observed for lesions. OD values were determined at 490 nm. The half-effective concentration (EC_50_), the half-toxic concentration of the drug (TC_50_), and the therapeutic index (TI) were calculated by the Reed-Muench method [23].

$$\mathrm{Distance}\;\mathrm{ratio}=\frac{{\mathrm P}_1-50\%}{{\mathrm P}_1-{\mathrm P}_2}$$

$${\mathrm{TC}}_{50\;}\left(50\%\;\mathrm t\mathrm o\mathrm x\mathrm i\mathrm c\;\mathrm c\mathrm o\mathrm n\mathrm c\mathrm e\mathrm n\mathrm t\mathrm r\mathrm a\mathrm t\mathrm i\mathrm o\mathrm n\right)=\left[\mathrm{Antilog}\;\left(\mathrm{logA}-\mathrm{distance}\;\mathrm{ratio}\right)\right]\;\times\;{\mathrm C}_{\mathrm m}$$

$${\mathrm{EC}}_{50}\left(50\%\;\mathrm e\mathrm f\mathrm f\mathrm e\mathrm c\mathrm t\mathrm i\mathrm v\mathrm e\;\mathrm c\mathrm o\mathrm n\mathrm c\mathrm e\mathrm n\mathrm t\mathrm r\mathrm a\mathrm t\mathrm i\mathrm o\mathrm n\right)=\left[\mathrm{Antilog}\;\left(\mathrm{logB}-\mathrm{distance}\;\mathrm{ratio}\right)\right]\;\times{\mathrm C}_{\mathrm m}$$

$$\mathrm{TI}\;\left(\mathrm{therapeutic}\;\mathrm{index}\right)=\frac{{\mathrm{TC}}_{50}}{{\mathrm{EC}}_{50}}$$

P_1_: The cytopathic rate which is more than 50%

P_2_: The cytopathic rate which is less than 50%

A: The dilution ratio of the drug added to the well which has a cytopathic rate greater than 50%

B: The dilution ratio of the drug added to the well which has a cell survival rate greater than 50%

C_m_: Initial concentration of drug

### Metabolomic sample preparation

Hep-2 cells were seeded in culture dishes at a density of 1 × 10^8^ cells/L and LJF was administered at the maximum nontoxic concentration and incubated for 24 h. Each dish was inoculated with 1 mL of RSV at 100 TCID_50_. Normal and model groups were set up simultaneously. Cells were collected and quenched with methanol and repeatedly frozen and thawed. Then, the samples were collected in 2 mL Eppendorf tubes and vortexed for 30 s. After standing at -40 ℃ for 1 h, the samples were centrifuged at 4 °C for 15 min at 12,000 rpm (13,800 × g, *r* = 8.6 cm). All samples were dried to the same volume (660 μL) and reconstituted with 100 μL of methanol: acetonitrile: water = 2: 2: 1 (containing isotopically labeled internal standard mixture). After that, the samples were vortexed for 30 s, sonicated in an ice bath for 10 min, and centrifuged for 15 min at 12,000 rpm at 4 °C. The supernatant was collected for LC–MS analysis.

### LC–MS analysis conditions

A UHPLC system (Vanquish, Thermo Fisher Scientific, USA) with a Waters ACQUITY UPLC BEH Amide (2.1 mm × 100 mm, 1.7 μm) coupled to a high-resolution mass spectrometer (Q Exactive HFX, Thermo Fisher Scientific, USA) was used for LC–MS analysis. The samples were analyzed on a Waters ACQUITY UPLC BEH Amide (2.1 mm × 100 mm, 1.7 μm) column by Vanquish UHPLC system. The injection volume was 2 μL. The mobile phase used aqueous solution containing 25 mmol/L ammonium acetate and 25 mmol/L ammonia (A), and acetonitrile (B).

The mass spectrometry was performed using an electrospray ionization (ESI) source in both positive and negative ion modes. The detailed parameters were set as follows: the sheath gas flow rate was 30 Arb, aux gas flow rate was 25 Arb, capillary temperature was 350 ℃, full MS resolution was 60000, MS/MS resolution was 7500, collision energy was 10/30/60 in NCE mode, spray Voltage was 3.6 kV (positive) or -3.2 kV (negative).

### Potential biomarker upstream protein PPI analysis

The MetScape 3.1.3 software was used to analyze the interaction relationship between the upstream target of differential metabolites and the anti-RSV target of LJF, and the PPI analysis was performed in STRING, and finally, the network was visualized by Cytoscape 3.7.1.

### Multivariate data processing of metabolomics

The collected LC–MS raw data files were imported into the ProteoWizard software and converted to mzXML format. Then, the R package was used for peak identification, extraction, alignment, integration, etc. The data were matched with the self-built secondary mass spectrometry database, BiotreeDB (V2.1), for substance annotation with an algorithm score cutoff of 0.3. The software SIMCA 16.0.2 was used for multivariate analysis of the data, such as orthogonal partial least squares discriminant analysis (OPLS-DA). The variable importance values (VIP) from the project were used to rank the overall contribution of each variable in the OPLS-DA [7]. VIP > 1, *p* value < 0.05, and Fold Change (FC) > 1.5 or < 0.667 were used to select potential biomarkers.

**Fig. 1:**
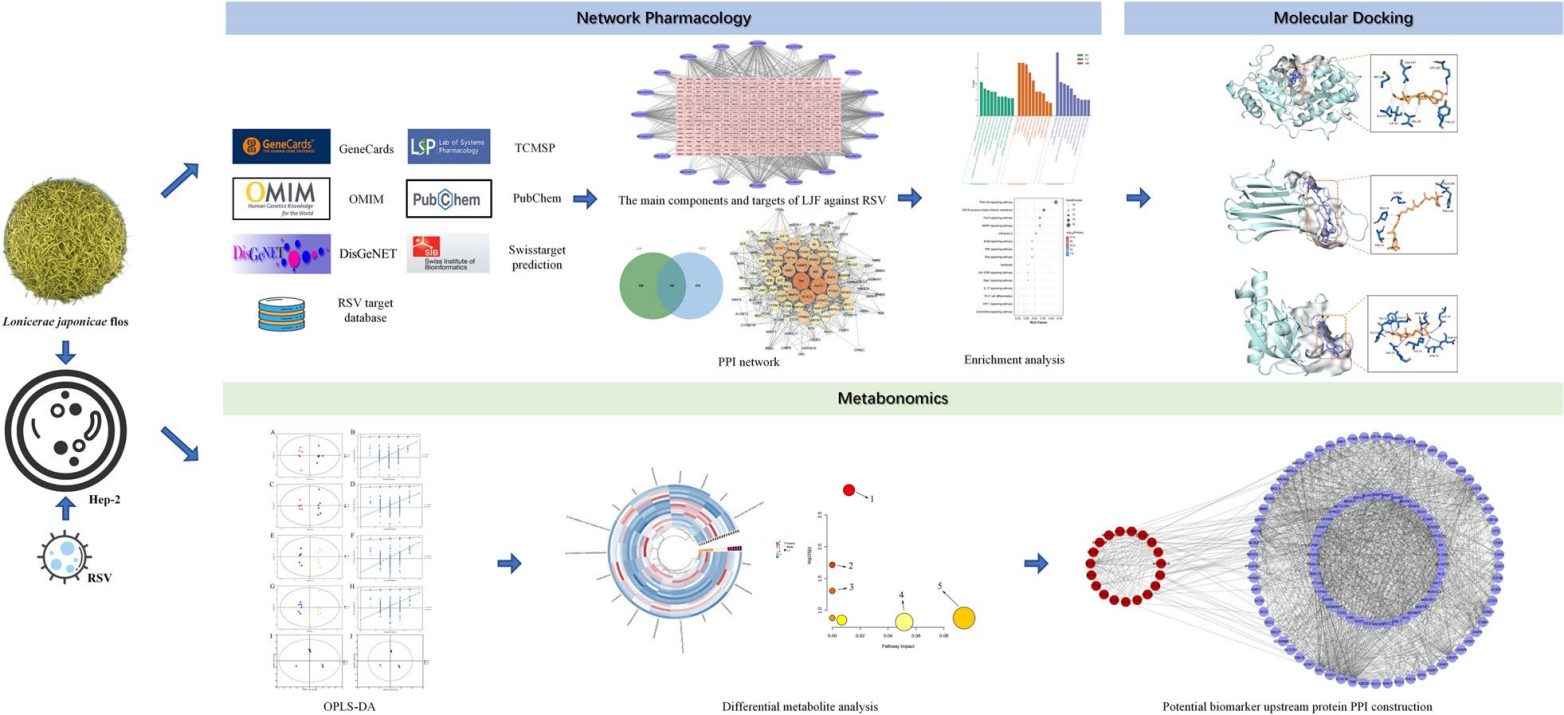
Flow chart of the design

Subsequently, the mass spectrometry information was matched with metabolite databases such as HMDB (http://www.hmdb.ca/) and METLIN (https://metlin.scripps.edu/) for feature peak identification. The mass spectrometry mass error was set to less than 10 ppm, and metabolites were identified based on the matching scores of the secondary mass spectrometry. The positive and negative ion mode data were merged into one data matrix table for subsequent analysis. The screened biomarkers were imported into the Gene Denovo platform (https://www.omicsshare.com/tools/home/report/report_circular_heatmap.html#) for heat map analysis, and normalization and clustering analysis were performed. Pathway enrichment analysis was performed using the MetaboAnalyst 5.0 platform (https://www.metaboanalyst.ca/), and the pathways with high impact were identified as relevant metabolic pathways. The process of the experiment is shown in Fig. 1.

## Results

### Prediction of LJF compounds and RSV disease targets

Through TCMSP search, 236 JLF chemical components were obtained. Based on the screening criteria, a total of 23 potential compounds of LJF against RSV were obtained from the TCMSP database (Table S1). A total of 1,267 potential compound-related targets of LJF were identified based on the TCMSP database, PubChem Server, Swiss Target Prediction (Table S2). By integrating data from GeneCard, DisGeNET, OMIM databases, and the RSV disease target database constructed by the research team, a total of 780 genes associated with RSV pneumonia were obtained (Table S3).

**Fig. 2:**
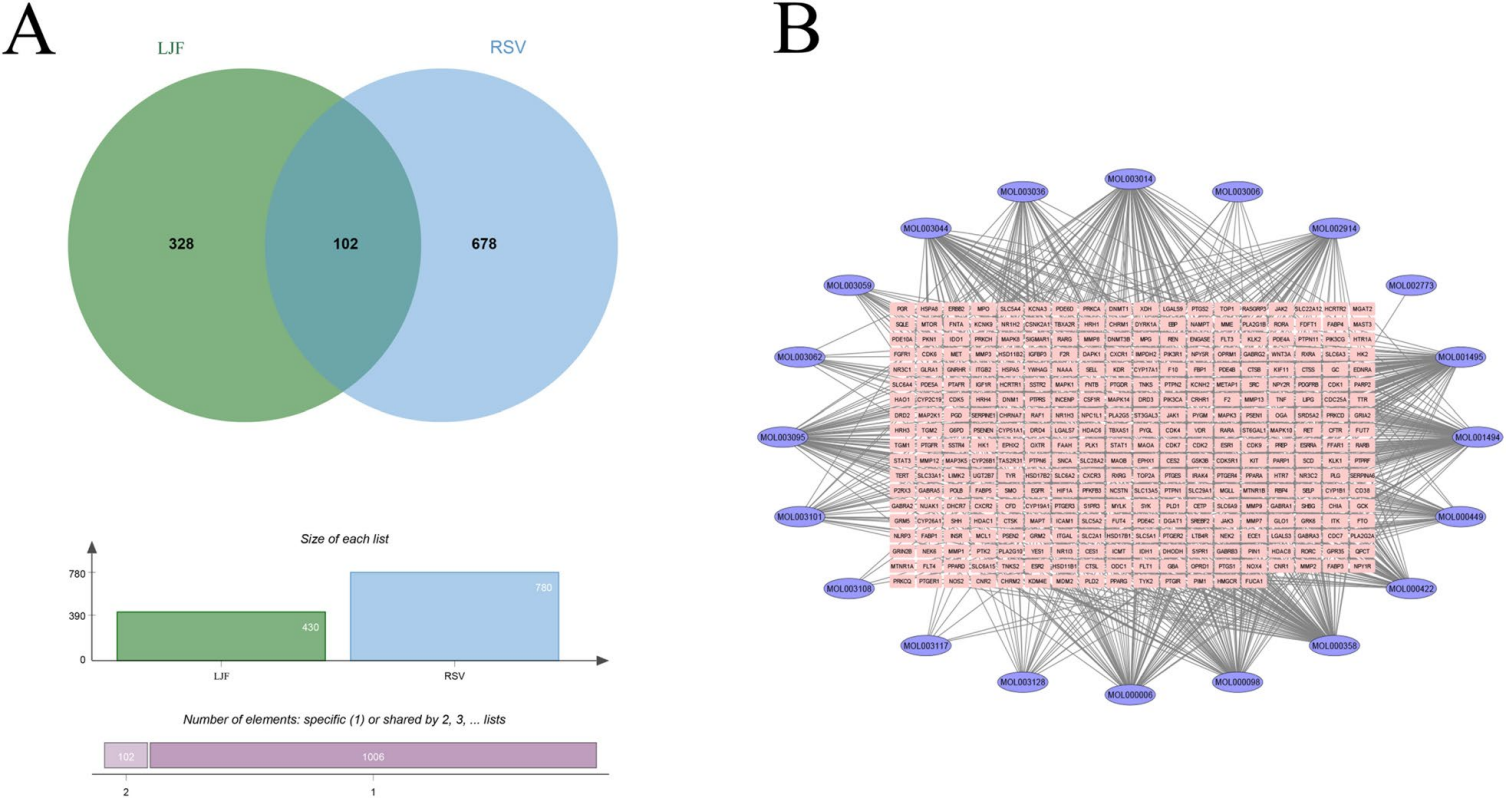
Network pharmacological analysis of the main components and targets of LJF against RSV. **A** LJF-RSV target mapping results; **B** LJF active ingredient-RSV pneumonia disease targets network map. The pink dots represent the active components of LJF, and the blue rectangular nodes represent the effective targets against RSV

After excluding false-positive targets and redundant targets, a total of 102 potential targets for LJF against RSV infection were identified (Fig. 2A). Details of targets are shown in Table S4. In addition, a network of "LJF active compounds-RSV pneumonia disease targets" was generated using Cytoscape 3.7.2 (Fig. 2B). It showed that beta-sitosterol, Ethyl linolenate, and Mandenol can regulate most of the targets, which may be the important components of LJF to exert its anti-RSV effect. The active ingredient of LJF-RSV pneumonia target network map can vividly reflect that traditional Chinese medicine is through a multi-component, multi-target disease network.

### PPI network analysis

**Fig. 3:**
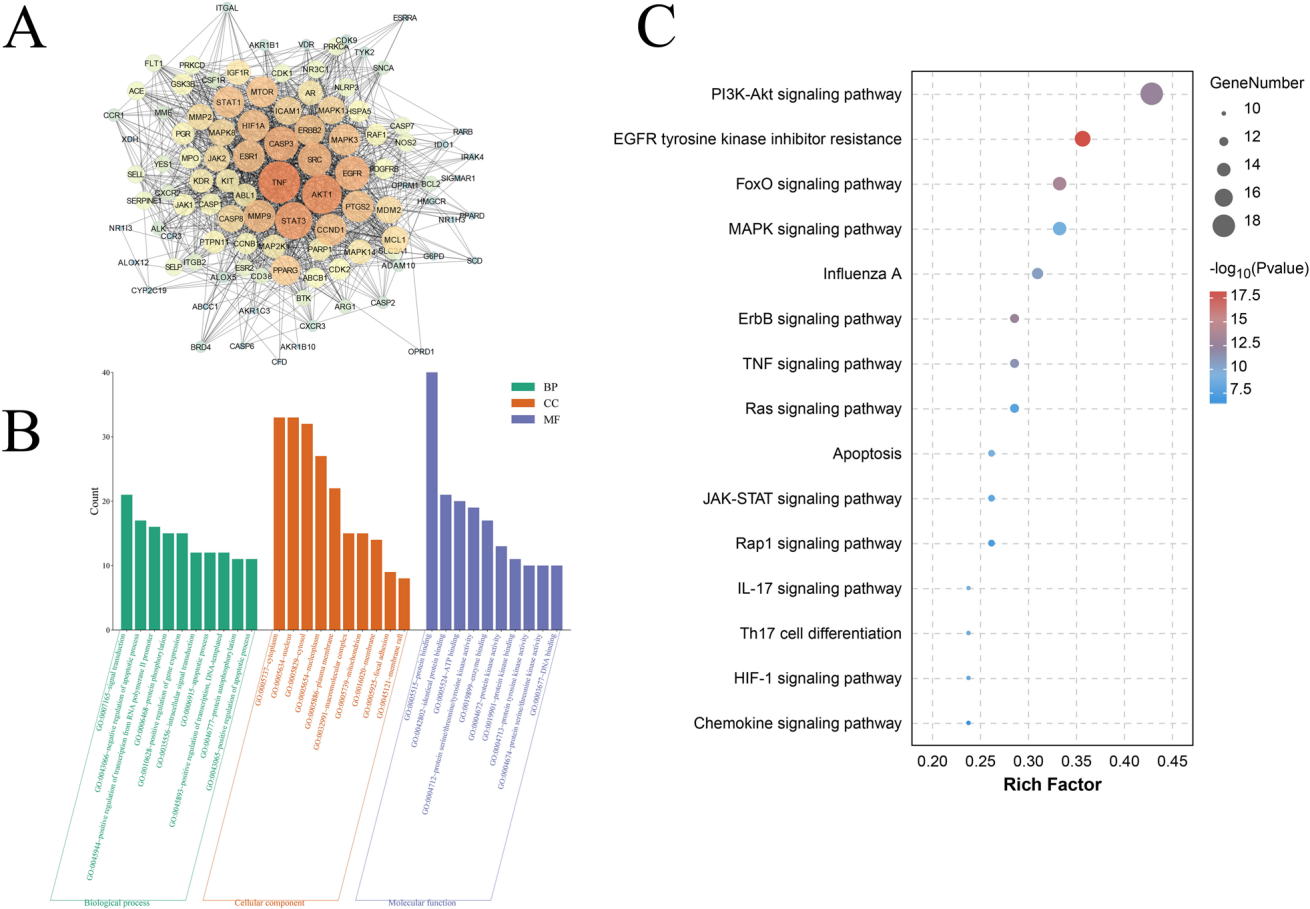
PPI network and enrichment analysis of anti-RSV target of LJF. **A** PPI network of "LJF-RSV"; **B** GO enrichment bar chart; **C** KEGG pathway enrichment bubble diagram. * *p* < 0.05

To identify the potential mechanisms of LJF against RSV, PPI was used to analyze the correlation between LJF and RSV targets. As shown in Fig. 3A, 102 potential therapeutic targets were constructed. Cytoscape 3.7.2 was used to screen and visualize the results of PPI and obtain the interaction network of the anti-RSV targets of LJF, which contained 102 nodes, 1358 edges, and an average degree value of 26.62. The importance of nodes is assessed based on the node value, the higher the node value, the larger the node, and the darker the node color, which means the node is more important. The protein–protein interaction network visualized the interaction between LJF active components and target genes, further revealing the synergistic anti-RSV effect of LJF through multi-component and multi-target.

### Go and KEGG pathway enrichment analyses

GO enrichment analysis was completed by David and the target proteins were analyzed. The results are shown in Fig. 3B, where CC, BP, and MF all show only the top 10 results with the largest count values. The results showed that CC was mainly related to the cytoplasm, nucleus, and cytosol. BP was mainly associated with signal transduction, negative regulation of apoptotic process, and positive regulation of transcription from RNA polymerase II promoter. MF was mainly linked with protein binding, identical protein binding, and ATP binding. From the GO functional analysis, it was indicated that these target proteins of LJF anti-RSV were associated with many biological processes and molecular functions.

In addition, KEGG pathway enrichment analysis showed (Fig. 3C) that LJF anti-RSV may be involved in signaling pathways such as PI3K-Akt signaling pathway, EGFR tyrosine kinase inhibitor resistance, FoxO signaling pathway, MAPK signaling pathway, and other pathways.

### Molecular docking of active ingredients in LJF with core target proteins

**Fig. 4:**
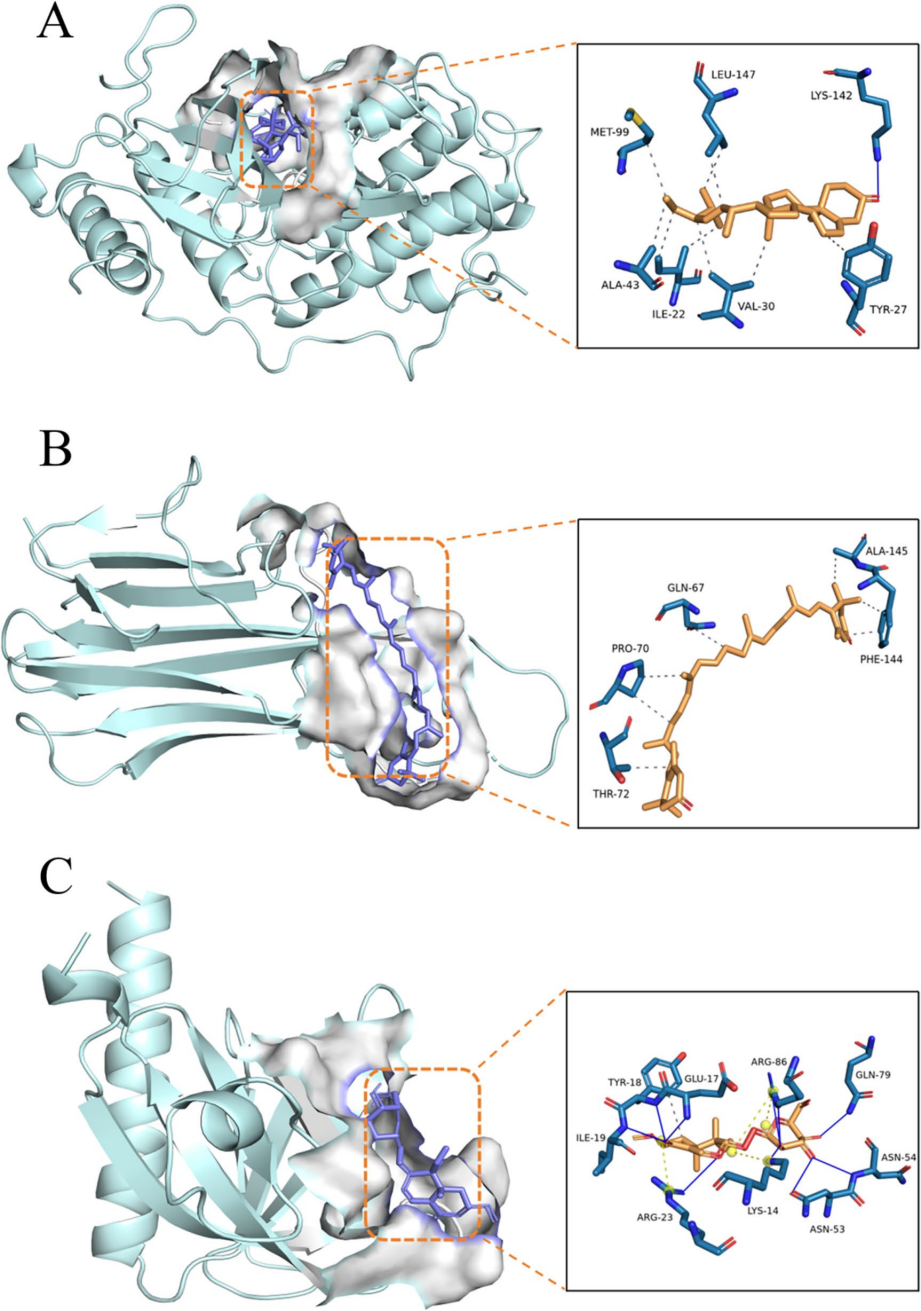
Docking results. **A** Docking between ZINC03978781 and STAT3, (**B**) Docking between 4,5'-Retro-.beta.,.beta.-Carotene -3,3'-dione, 4',5'-didehydro- and TNF, and (**C**) Docking between 7-epi-Vogeloside and AKT1

To further investigate the potential interactions between the active ingredients in LJF and the core target proteins, we performed molecular docking analyses to validate the binding ability of the selected active ingredients MOL00306 (ZINC03978781) with STAT3, MOL003062 (4,5'-Retro-.beta.,.beta.-Carotene -3,3'-dione, 4',5'-didehydro-) with TNF, and MOL003101 (7-epi-Vogeloside) with AKT1. The components with lower binding energy were screened (Table 1 and Fig. 4). In general, lower binding energy indicates stronger binding affinity, more stable conformation, and potentially stronger interaction. For STAT3 as shown in Fig. 4A, MOL003036 (ZINC03978781) could respectively form a hydrogen bond with LYS-142; and a hydrophobic interaction with LEU-147, MET-99, ALA-43, ILE-22, VAL-30 and TYR-27. For TNF as shown in Fig. 4B, MOL003062 (4,5'-Retro-.beta.,.beta.-Carotene -3,3'-dione, 4',5'-didehydro-) could respectively form a hydrophobic interaction with THR-72, PRO-70, GLN-67, ALA-145 and PHE-144. For AKT1 as shown in Fig. 4C, MOL003101 (7-epi-Vogeloside) could respectively form a hydrogen bond with GLU-17, TYR-18, ILE-19, ARG-23, LYS-14, ASN-53, ASN-54, GLN-79 and ARG-86; a hydrophobic interaction with TYR-18; and a Charge Center with ARG-86, LYS-14 and ARG-23.

**Table 1:** Binding energy results from molecular docking

Target	Molecule	Types of Bonds	Residues	Binding Energy (kcal/mol)
STAT3	ZINC03978781	Hydrogen Bond	LYS-142	-8.5
		Hydrophobic Interaction	LEU-147, MET-99, ALA-43, ILE-22, VAL-30, TYR-27	
TNF	4,5'-Retro-.beta.,.beta.-Carotene -3,3'-dione, 4',5'-didehydro-	Hydrophobic Interaction	THR-72, PRO-70, GLN-67, ALA-145, PHE-144	-7.6
AKT1	7-epi-Vogeloside	Hydrogen Bond	GLU-17, TYR-18, ILE-19, ARG-23, LYS-14, ASN-53, ASN-54, GLN-79, ARG-86	-6.4
		Hydrophobic Interaction	TYR-18	
		Charge Center	ARG-86, LYS-14, ARG-23

### Inhibitory effect of LJF on RSV

The OD value was determined by MTT, and the calculated TC_50_ value of LJF was 5.37 (mg/mL), the EC_50_ value was 1.12 (mg/mL), and the TI value was 4.79. Therefore, it can be concluded that LJF has some in vitro inhibitory activity against RSV.

### Metabolomics analysis

**Fig. 5:**
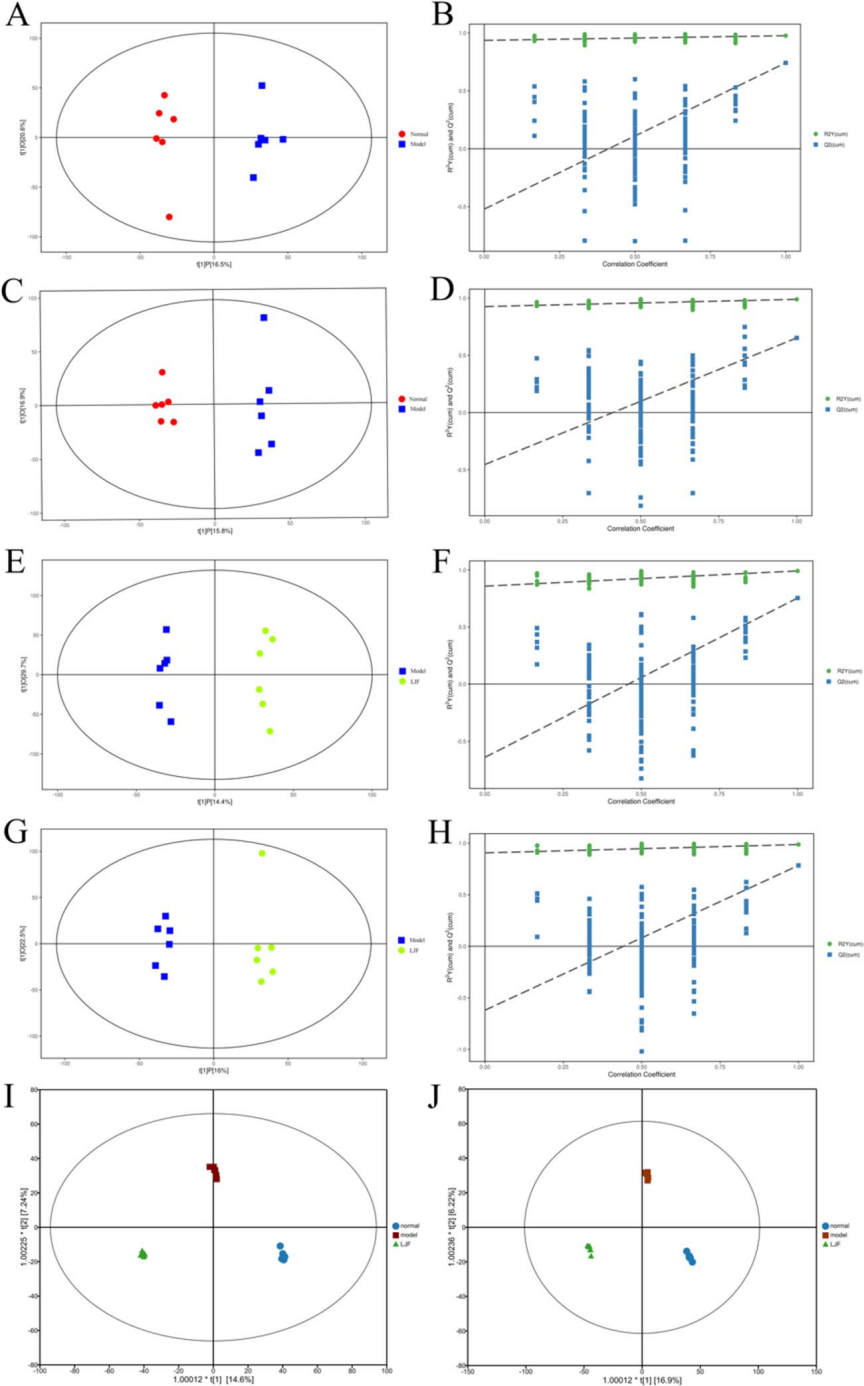
The results of OPLS-DA score plots and 200-permutation tests. **A**, **B** OPLS-DA score plot and 200-permutation tests for normal and model group in positive ion mode (R2X = 0.371, R2Y = 0.976, Q2 = 0.742); **C**, **D** OPLS-DA score plot and 200-permutation tests for normal and model group in negative ion mode (R2X = 0.327, R2Y = 0.989, Q2 = 0.652); **E**, **F** OPLS-DA score plot and 200-permutation tests for model and LJF group in positive ion mode (R2X = 0.441, R2Y = 0.992, Q2 = 0.755); **G**, **H** OPLS-DA score plot and 200-permutation tests for model and LJF group in negative ion mode (R2X = 0.386, R2Y = 0.987, Q2 = 0.784). **I** OPLS-DA score plot for the three groups together in positive ion mode (R2X = 0.614, R2Y = 0.995, Q2 = 0.713). **J** OPLS-DA score plot for the three groups together in negative ion mode (R2X = 0.611, R2Y = 0.996, Q2 = 0.712)

To investigate the metabolic effects of LJF on RSV-infected Hep-2 cells, OPLS-DA analysis was performed. The results showed that the model group was differentiated from the control group at the 95% confidence interval, indicating successful modeling. In addition, there was also a significant distinction between the model group and the LJF-treated group in positive and negative ion mode, indicating that LJF intervention had a significant modulating effect on RSV infection (Fig. 5A, C, E, G). To better demonstrate the relationship between the three groups in the comparison, we superimposed the OPLS-DA score scatter plot for the three groups (Fig. 5I, J). The predictive ability of the OPLS-DA model (Fig. 5B, D, F, H) was verified using the permutation test (*n* = 200), and the experimental values of R^2^, representing the goodness-of-fit of the model, and Q^2^, representing the predictive ability of the model, on the left side were consistently lower than the original values on the right side. This indicated that the results were not overfitted and that OPLS-DA had better predictive ability. These analyses demonstrate the statistical validity of the separation model and its ability to explain the differences between the two sample groups.

### Differential metabolite selection

Based on VIP values, FC, and *p*-values derived from the OPLS-DA model, a comparative analysis was performed among the three groups to select potential metabolites contributing significantly to clustering and discrimination. Ultimately, 18 differential metabolites were identified, including phospholipids, nucleosides, and choline compounds (Table 2). The detailed information includes the chemical name, relevance score, m/z, and HMDB, among others.

**Table 2:** Common biomarkers of three groups in positive and negative ion mode

ID	Metabolite	Score	FC	RT	MZ	HMDB	Model/Normal	LJF/Model
1	5-Aminopentanal	0.982238	2.245	96.2756	102.0915	0012815	down	up
2	( +)-2,3-Dihydro-3-methyl-1H-pyrrole	0.979322	2.009	95.5122	84.08106	0033529	down	up
3	N,2,3-Trimethyl-2-(1-methylethyl)butanamide	0.956896	2.259	239.1300	172.1693	0036195	down	up
4	(2S,4R,5S)-Muscarine	0.905261	2.328	253.6780	174.1488	0029936	down	up
5	Triethanolamine	0.895523	5.852	179.1160	150.1123	0032538	down	up
6	7-Dehydrocholesterol	0.871764	2.052	82.9849	385.3414	0000032	down	up
7	Koeniginequinone A	0.806357	1.669	426.7350	242.0789	0032085	down	up
8	3b,18b-3-Methoxy-11-oxo-12-oleanen-30-oic acid	0.775299	1.829	216.4950	485.3577	0034516	down	up
9	d-Tocotrienol	0.714779	1.645	246.9855	397.3053	0030008	down	up
10	Adenosine	0.602982	0.454	185.6020	268.1034	0000050	up	down
11	PC(22:4(7Z,10Z,13Z,16Z)/14:1(9Z))	0.552080	0.522	173.2545	780.5398	0008624	up	down
12	N(6)-(Octanoyl)lysine	0.550383	15.607	69.5296	273.2171	0011684	down	up
13	Santene	0.459449	2.347	96.2827	123.1168	0038140	down	up
14	Carbadox	0.895639	5.282	359.234	261.0615	0031762	down	up
15	Deoxyinosine	0.875144	0.353	196.702	251.0787	0000071	up	down
16	Deoxyguanosine	0.855528	0.274	252.174	266.0897	0000085	up	down
17	Cosmosiin	0.800533	1.916	23.2723	431.0963	0037340	down	up
18	Cytidine	0.489911	1.627	407.657	242.0801	0000089	down	up

### Differential metabolite analysis

#### Cluster analysis

**Fig. 6:**
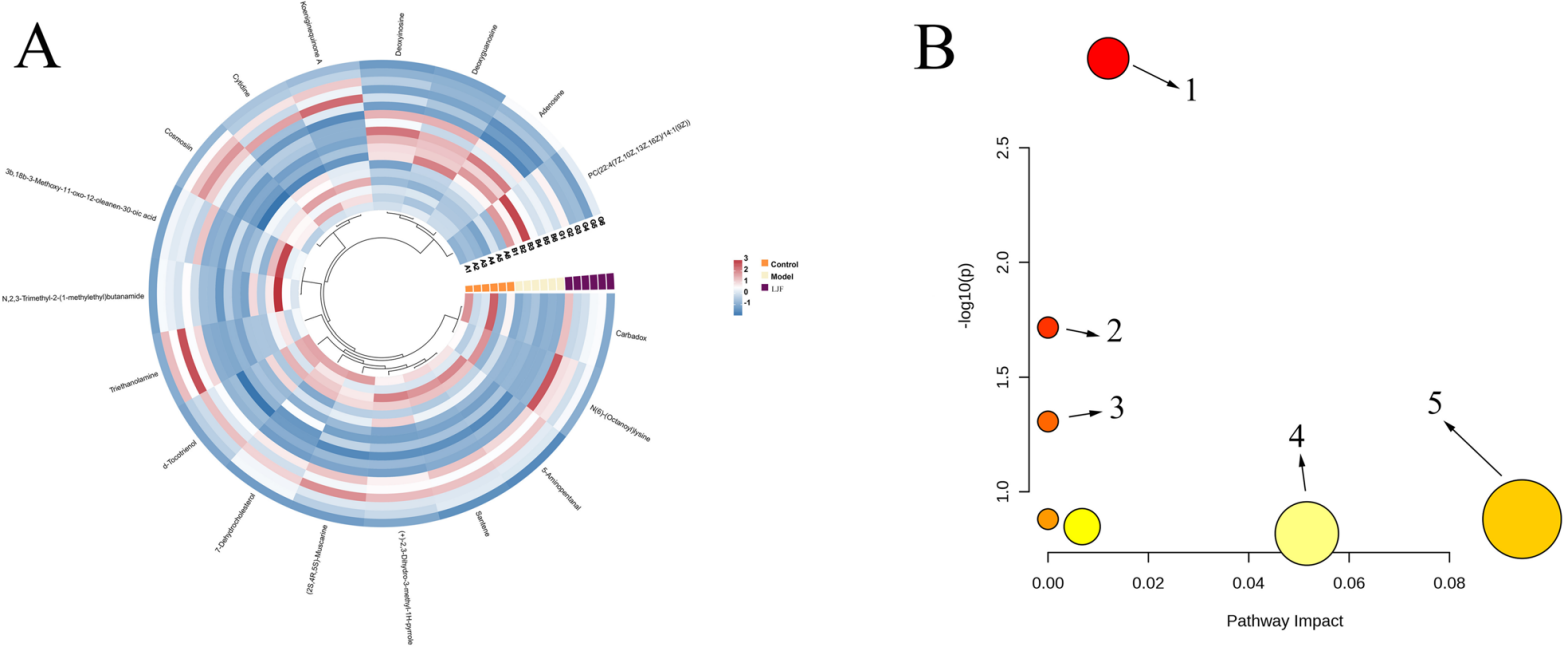
Differential metabolite analysis. **A** the expression hot map of biomarkers represents the difference in the content of metabolic markers of LJF intervention in RSV infection. Note: the color depth reflects the value of the variable. **B** metabolic pathway related to anti-RSV infection of LJF. Note: 1. Purine metabolism; 2. Linoleic acid metabolism; 3. alpha-Linolenic acid metabolism; 4. Steroid biosynthesis; 5. Glycerophospholipid metabolism

Hierarchical cluster analysis was employed to investigate the effects of LJF on RSV infection by clustering the differential metabolites. The results revealed significant differences between the control group and the model group. In contrast, the LJF group was able to restore the levels of differential metabolites to normal, indicating that LJF treatment can alleviate metabolic disturbances induced by RSV infection (Fig. 6A).

#### Metabolic pathway analysis

To investigate the metabolic pathways of potential biomarkers of LJF anti-RSV, MetaboAnalyst 5.0 was used to analyze the pathway enrichment of potential biomarkers. The results showed (Fig. 6B) that five important pathways, including purine metabolism, linoleic acid metabolism, alpha-linolenic acid metabolism, steroid biosynthesis, and glycerophospholipid metabolism, played critical roles in the regulation of LJF against RSV infection.

### Potential biomarker upstream protein PPI construction

**Fig. 7:**
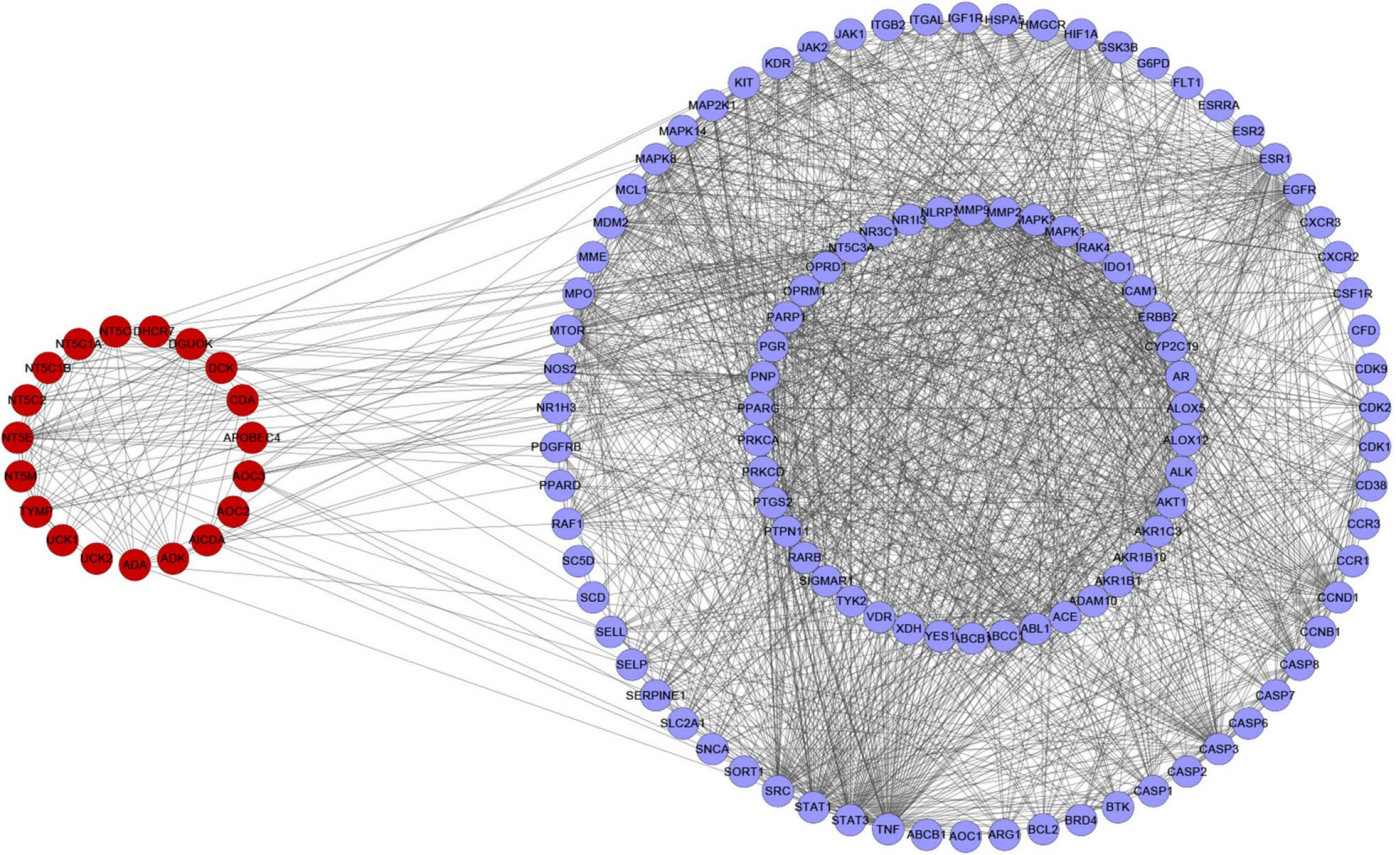
Potential biomarker upstream protein PPI network. Red and blue nodes represent metabolite-associated targets and LJF anti-RSV-associated targets, respectively

To clarify the potential mechanism of LJF anti-RSV further, the correlation between metabolic pathways and corresponding targets was further analyzed. MetScape collected 19 upstream proteins based on the relevant metabolic targets, and subsequently generated PPI relationships with LJF anti-RSV targets, as shown in Fig. 7. The network has 127 nodes and 1513 edges, with more edges indicating stronger interactions. Based on this network, a link between upstream targets and disease targets can be found, which indirectly proves the results of metabolomics [7].

## Discussion

RSV is one of the pathogens which cause lower respiratory tract inventions and is still a significant cause of death in infants and elderly patients worldwide [1]. Its incidence is high and no effective vaccine has been developed [24, 25]. The only RSV prevention drug currently approved by the Food and Drug Administration (FDA) is palivizumab, but its stringent vaccination requirements and high cost have limited its large-scale use [26]. Traditional Chinese herbs are gaining attention as promising complementary and alternative therapies to fight infections [27]. LJF has been used for many years in China for its efficacy in the treatment of bacterial and viral infections, as documented in *Mingyi Bielu* [8, 9, 28]. LJF was the most popular traditional Chinese medicine used in treatments of severe acute respiratory syndromes (SARS) and influenza A in 2003 and 2009 [29]. Research by Gao et al. [30] showed that LJF extracts exerted protective effects against Hydroquinone-induced cytotoxicity in hepatic L02 cells. Xie et al. [9] explored the quality evaluation method of LJF by combining anti-H1N1 activity with chemical pattern recognition, which is of great significance for ensuring the clinical effect of LJF antiviral. Zeng et al. [6] reviewed several anti-RSV efficacies of LJF. It may be an economical and promising therapeutic agent for RSV, but its underlying pharmacological mechanism is not fully understood. This study aimed to investigate the possible mechanism of action of LJF against RSV through the combination of network pharmacology and metabolomics.

Through the network pharmacology study, we successfully screened 338 potential anti-RSV targets and 20 components of LJF. Then, we constructed PPI networks of related targets and found that STAT3, TNF, and AKT1 might be the key targets that play a role. By performing KEGG pathway analysis on the targets, we revealed that LJF may be anti-RSV by modulating relevant targets on the PI3K-Akt signaling pathway, EGFR tyrosine kinase inhibitor resistance, and FoxO signaling pathway. Molecular docking revealed the interaction patterns of STAT3 with ZINC03978781, TNF with 4,5'-Retro-.beta.,.beta.-Carotene-3,3'-dione, 4',5'-didehydro-, and AKT1 with 7-epi-Vogeloside. This result provides a basis for the molecular mechanism of the anti-RSV activity of LJF and provides a direction for further research.

In addition, metabolomics analysis also provided us with important information about the anti-RSV effects of LJF. By LC–MS combined with multivariate data processing, we found that LJF may regulate 18 potential biomarkers such as Adenosine, PC (22:4(7Z,10Z,13Z,16Z)/14:1(9Z)). These biomarkers involved metabolic pathways such as Purine metabolism and Linoleic acid metabolism, suggesting that LJF may play a role in the anti-RSV process by influencing the regulation of metabolic pathways. This finding further enriches our understanding of the mechanism of LJF's anti-RSV action.

Viral replication requires the integration of a great number of nucleoside analogs, so the activity of viral RNA-dependent RNA Polymerase (RdRp) can be affected by regulating the expression level of metabolites such as adenosine, which in turn can play an anti-RSV role [31]. For example, the broad-spectrum RNA virus inhibitor favipiravir (T-705) was approved for marketing in Japan in 2014, and similarly, nucleoside analogs such as remdesivir are under investigation [32]. In the present study, significant differences in adenosine were found in the RSV-infected group, and its level was significantly regressed by the intervention of LJF [33]. Adenosine receptor antagonists have been reported to inhibit the production of pro-inflammatory cytokines (e.g., TNF-α, IL-1, and IL-6) by suppressing PKA activity and cAMP levels [34]. N6-methyladenosine (m6A) methylates and modifies the RSV mRNA/antigenome, which promotes genome replication, mRNA transcription, and viral protein synthesis and the production of zygotic infectious particles, whereas the removal of m6A reduces the amount of RSV [35]. Therefore, adenosine is important for viral replication and regulation of innate immunity, and it is hypothesized that LJF may exert anti-RSV replication and immunomodulatory effects by regulating nucleoside metabolism.

Lung surface active substances account for about 90% of lipids, of which PC is the most abundant and has some innate immunoreactivity [36, 37]. In this study, we found that the metabolism of intracellular PC (22:4(7Z,10Z,13Z,16Z)/14:1(9Z)) was disturbed after RSV infection, which could be significantly regulated and brought to normal levels after treatment with LJF. Du et al. [38] found that the levels of several glycerophospholipids were significantly down-regulated after RSV infection, and most of the indexes tended to recover to the normal group after administration of Jinxin Oral Liquid, which is consistent with the results of the present study. In addition, down-regulation of phospholipid metabolism can enhance processes such as autophagy or *β*-oxidation, and catabolism to produce ATP and inhibit fatty acid synthesis, thus limiting RNA virus replication [39].

Overall, the network pharmacology and metabolomics studies indicated that LJF may exert its anti-RSV effects by modulating the PI3K-Akt signaling pathway, EGFR tyrosine kinase inhibitor resistance, and other related pathways, by ZINC03978781, 4,5'-Retro-.beta.,.beta.-Carotene -3,3'-dione, 4',5'-didehydro and 7-epi-Vogeloside interfering with the targets such as STAT3, TNF, and AKT1, as well as affecting the metabolic pathways such as adenosine and phospholipid metabolism. The results provide important clues for further investigation of the anti-RSV mechanism of LJF and theoretical support for its use as a cost-effective anti-RSV therapeutic agent. However, this study still has certain limitations. Firstly, it is challenging to generalize the dose–response relationship between drugs and diseases. Secondly, a more in-depth investigation and validation of the specific mechanisms of LJF are required to provide more reliable evidence for clinical applications.

## Conclusion

This study employed metabolomics and network pharmacology to systematically analyze the potential targets, pathways, and metabolites of LJF in the anti- RSV context. The results revealed that components such as ZINC03978781, 4,5'-Retro-.beta.,.beta.-Carotene -3,3'-dione, 4',5'-didehydro, and 7-epi-Vogeloside effectively modulate the expression of STAT3, TNF, and AKT1 targets, thereby contributing to the therapeutic effects against RSV infection. Metabolomic findings demonstrated the regulatory effects of 18 biomarkers and their metabolic pathways, actively participating in LJF's anti-RSV actions. The outcomes of this study provide theoretical support for the clinical application of LJF in combating RSV and establish a foundation for its further widespread use.

### Supplementary Information

**Additional file 1:** **Supplementary Table S1.** LJF active components.

**Additional file 2:** **Supplementary Table S2.** Corresponding targets of active constituents.

**Additional file 3:** **Supplementary Table S3.** RSV infection-related genes.

**Additional file 4:** **Supplementary Table S4.** Potential target of LJF against RSV infection.

## Data Availability

All data in this study are included in this article and its supplementary information files.

## Abbreviations

RSV: Respiratory Syncytial Virus
LJF: *Lonicerae japonicae* Flos
SARS-COV-2: Severe acute respiratory syndrome coronavirus
PPI: Protein–Protein Interaction
GO: Gene Ontology
KEGG: Kyoto Encyclopedia of Genes and Genomes
CC: Cell component
BP: Biological process
MF: Molecular function
Hep-2: Human laryngeal cancer cell line
TCMSP: Traditional Chinese Medicine Systems Pharmacology Database
OB: Oral bioavailability
DL: Drug-likeness
EC_50_: Half-effective concentration
TC_50_: Half-toxic concentration of the drug
TI: Therapeutic index
ESI: Electrospray ionization
OPLS-DA: Orthogonal partial least squares discriminant analysis
VIP: Variable importance values
FC: Fold Change
FDA: Food and Drug Administration
RdRp: RNA-dependent RNA Polymerase
m6A: N6-methyladenosine
