# Supplementary material for: Network pharmacology combined with metabolomics to explore the mechanism for Lonicerae Japonicae flos against respiratory syncytial virus
Source: BMC Complement Med Ther. 2023 Dec 12;23:449. doi: 10.1186/s12906-023-04286-0 (PMC10714634; doi:10.1186/s12906-023-04286-0)
Supplement: Supplementary file 1 — Additional file 1: Supplementary Table S1. LJF active components. [file 12906_2023_4286_MOESM1_ESM.docx]

**Network pharmacology combined with metabolomics to explore the mechanism for *Lonicerae japonicae* flos against Respiratory Syncytial Virus**

Jie Ding^1^, Jing Li^1^, Zhe Zhang^1^, Yaxuan Du^2^, Yuhong Liu^1, *^, Ping Wang^3, *^, Haitao Du^3, *^

^1^ College of Pharmacy, Shandong University of Traditional Chinese Medicine, Jinan, 250355, China

^2^ School of Chinese Materia Medica, Shenyang Pharmaceutical University, Shenyang 117004, China

^3^ Shandong Academy of Chinese Medicine, Jinan, 250014, China

^*^Corresponding author. Yuhong Liu, Shandong University of Traditional Chinese Medicine, Jinan, 250355, China. Ping Wang and Haitao Du, Shandong Academy of Chinese Medicine, Jinan, 250014, China.

E-mail addresses: liuyuhongwu@126.com (Yuhong Liu), wangpingjinan@126.com (Ping Wang), kkitdht@foxmail.com (Haitao Du).

**Supplementary Table S1. LJF active components**

| Mol ID | Molecule Name | OB (%) | DL |
| --- | --- | --- | --- |
| MOL001494 | Mandenol | 42.00 | 0.19 |
| MOL001495 | Ethyl linolenate | 46.10 | 0.20 |
| MOL002707 | phytofluene | 43.18 | 0.50 |
| MOL002914 | Eriodyctiol (flavanone) | 41.35 | 0.24 |
| MOL003006 | (-)-(3R,8S,9R,9aS,10aS)-9-ethenyl-8-(beta-D-glucopyranosyloxy)-2,3,9,9a,10,10a-hexahydro-5-oxo-5H,8H-pyrano[4,3-d]oxazolo[3,2-a]pyridine-3-carboxylic acid_qt | 87.47 | 0.23 |
| MOL003014 | secologanic dibutylacetal_qt | 53.65 | 0.29 |
| MOL002773 | beta-carotene | 37.18 | 0.58 |
| MOL003036 | ZINC03978781 | 43.83 | 0.76 |
| MOL003044 | Chryseriol | 35.85 | 0.27 |
| MOL003059 | kryptoxanthin | 47.25 | 0.57 |
| MOL003062 | 4,5'-Retro-.beta.,.beta.-Carotene-3,3'-dione, 4',5'-didehydro- | 31.22 | 0.55 |
| MOL003095 | 5-hydroxy-7-methoxy-2-(3,4,5-trimethoxyphenyl)chromone | 51.96 | 0.41 |
| MOL003101 | 7-epi-Vogeloside | 46.13 | 0.58 |
| MOL003108 | Caeruloside C | 55.64 | 0.73 |
| MOL003111 | Centauroside_qt | 55.79 | 0.50 |
| MOL003117 | Ioniceracetalides B_qt | 61.19 | 0.19 |
| MOL003124 | XYLOSTOSIDINE | 43.17 | 0.64 |
| MOL003128 | dinethylsecologanoside | 48.46 | 0.48 |
| MOL000358 | beta-sitosterol | 36.91 | 0.75 |
| MOL000422 | kaempferol | 41.88 | 0.24 |
| MOL000449 | Stigmasterol | 43.83 | 0.76 |
| MOL000006 | luteolin | 36.16 | 0.25 |
| MOL000098 | quercetin | 46.43 | 0.28 |
