# Supplementary material for: Network pharmacology combined with metabolomics to explore the mechanism for Lonicerae Japonicae flos against respiratory syncytial virus
Source: BMC Complement Med Ther. 2023 Dec 12;23:449. doi: 10.1186/s12906-023-04286-0 (PMC10714634; doi:10.1186/s12906-023-04286-0)
Supplement: Supplementary file 2 — Additional file 2: Supplementary Table S2. Corresponding targets of active constituents. [file 12906_2023_4286_MOESM2_ESM.docx]

**Network pharmacology combined with metabolomics to explore the mechanism for *Lonicerae japonicae* flos against Respiratory Syncytial Virus**

Jie Ding^1^, Jing Li^1^, Zhe Zhang^1^, Yaxuan Du^2^, Yuhong Liu^1, *^, Ping Wang^3, *^, Haitao Du^3, *^

^1^ College of Pharmacy, Shandong University of Traditional Chinese Medicine, Jinan, 250355, China

^2^ School of Chinese Materia Medica, Shenyang Pharmaceutical University, Shenyang 117004, China

^3^ Shandong Academy of Chinese Medicine, Jinan, 250014, China

^*^Corresponding author. Yuhong Liu, Shandong University of Traditional Chinese Medicine, Jinan, 250355, China. Ping Wang and Haitao Du, Shandong Academy of Chinese Medicine, Jinan, 250014, China.

E-mail addresses: liuyuhongwu@126.com (Yuhong Liu), wangpingjinan@126.com (Ping Wang), kkitdht@foxmail.com (Haitao Du).

**Supplementary Table S2. Corresponding targets of active constituents**

| MOL ID | Molecule Name | Target | UniProt ID | RSV resistant |
| --- | --- | --- | --- | --- |
| MOL000006 | luteolin | NOX4 | Q9NPH5 | √ |
| MOL000006 | luteolin | CDK5R1 | Q15078 | √ |
| MOL000006 | luteolin | CDK5 | Q00535 | √ |
| MOL000006 | luteolin | XDH | P47989 | √ |
| MOL000006 | luteolin | MAOA | P21397 | √ |
| MOL000006 | luteolin | FLT3 | P36888 | √ |
| MOL000006 | luteolin | CDK1 | P06493 | √ |
| MOL000006 | luteolin | GLO1 | Q04760 | √ |
| MOL000006 | luteolin | SYK | P43405 | √ |
| MOL000006 | luteolin | GSK3B | P49841 | √ |
| MOL000006 | luteolin | PARP1 | P09874 | √ |
| MOL000006 | luteolin | TTR | P02766 | √ |
| MOL000006 | luteolin | MMP9 | P14780 | √ |
| MOL000006 | luteolin | MMP2 | P08253 | √ |
| MOL000006 | luteolin | MMP12 | P39900 | √ |
| MOL000006 | luteolin | CD38 | P28907 | √ |
| MOL000006 | luteolin | CYP1B1 | Q16678 | √ |
| MOL000006 | luteolin | TNKS2 | Q9H2K2 | √ |
| MOL000006 | luteolin | TNKS | O95271 | √ |
| MOL000006 | luteolin | TOP1 | P11387 | √ |
| MOL000006 | luteolin | PTPRS | Q13332 | √ |
| MOL000006 | luteolin | HSD17B1 | P14061 | √ |
| MOL000006 | luteolin | CDK6 | Q00534 | √ |
| MOL000006 | luteolin | HSD17B2 | P37059 | √ |
| MOL000006 | luteolin | CYP19A1 | P11511 | √ |
| MOL000006 | luteolin | ESR2 | Q92731 | √ |
| MOL000006 | luteolin | CSNK2A1 | P68400 | √ |
| MOL000006 | luteolin | ESR1 | P03372 | √ |
| MOL000006 | luteolin | PTGS2 | P35354 | √ |
| MOL000006 | luteolin | CFTR | P13569 | √ |
| MOL000006 | luteolin | GRK6 | P43250 | √ |
| MOL000006 | luteolin | CDK2 | P24941 | √ |
| MOL000006 | luteolin | TERT | O14746 | √ |
| MOL000006 | luteolin | CDK1 | P06493 | √ |
| MOL000006 | luteolin | TYR | P14679 | √ |
| MOL000006 | luteolin | ESRRA | P11474 | √ |
| MOL000006 | luteolin | GPR35 | Q9HC97 | √ |
| MOL000006 | luteolin | IGF1R | P08069 | √ |
| MOL000006 | luteolin | EGFR | P00533 | √ |
| MOL000006 | luteolin | F2 | P00734 | √ |
| MOL000006 | luteolin | PIM1 | P11309 | √ |
| MOL000006 | luteolin | DRD4 | P21917 | √ |
| MOL000006 | luteolin | MPO | P05164 | √ |
| MOL000006 | luteolin | PIK3R1 | P27986 | √ |
| MOL000006 | luteolin | DAPK1 | P53355 | √ |
| MOL000006 | luteolin | PYGL | P06737 | √ |
| MOL000006 | luteolin | SRC | P12931 | √ |
| MOL000006 | luteolin | PTK2 | Q05397 | √ |
| MOL000006 | luteolin | KDR | P35968 | √ |
| MOL000006 | luteolin | MMP13 | P45452 | √ |
| MOL000006 | luteolin | MMP3 | P08254 | √ |
| MOL000006 | luteolin | PLK1 | P53350 | √ |
| MOL000006 | luteolin | PKN1 | Q16512 | √ |
| MOL000006 | luteolin | MET | P08581 | √ |
| MOL000006 | luteolin | NEK2 | P51955 | √ |
| MOL000006 | luteolin | CXCR1 | P25024 | √ |
| MOL000006 | luteolin | NEK6 | Q9HC98 | √ |
| MOL000006 | luteolin | PLA2G1B | P04054 | √ |
| MOL000006 | luteolin | NUAK1 | O60285 | √ |
| MOL000006 | luteolin | PFKFB3 | Q16875 | √ |
| MOL000006 | luteolin | PLG | P00747 | √ |
| MOL000006 | luteolin | KDM4E | B2RXH2 | √ |
| MOL000006 | luteolin | AKR1B1 | P15121 | × |
| MOL000006 | luteolin | CA2 | P00918 | × |
| MOL000006 | luteolin | CCNB3 | Q8WWL7 | × |
| MOL000006 | luteolin | CCNB1 | P14635 | × |
| MOL000006 | luteolin | CCNB2 | O95067 | × |
| MOL000006 | luteolin | ALOX5 | P09917 | × |
| MOL000006 | luteolin | ADORA1 | P30542 | × |
| MOL000006 | luteolin | CA7 | P43166 | × |
| MOL000006 | luteolin | APP | P05067 | × |
| MOL000006 | luteolin | CA12 | O43570 | × |
| MOL000006 | luteolin | CA4 | P22748 | × |
| MOL000006 | luteolin | ABCG2 | Q9UNQ0 | × |
| MOL000006 | luteolin | AKR1B10 | O60218 | × |
| MOL000006 | luteolin | ARG1 | P05089 | × |
| MOL000006 | luteolin | ABCC1 | P33527 | × |
| MOL000006 | luteolin | ACHE | P22303 | × |
| MOL000006 | luteolin | ABCB1 | P08183 | × |
| MOL000006 | luteolin | ADORA2A | P29274 | × |
| MOL000006 | luteolin | ALOX15 | P16050 | × |
| MOL000006 | luteolin | ALOX12 | P18054 | × |
| MOL000006 | luteolin | AMY1A | P04745 | × |
| MOL000006 | luteolin | CA1 | P00915 | × |
| MOL000006 | luteolin | CA9 | Q16790 | × |
| MOL000006 | luteolin | AHR | P35869 | × |
| MOL000006 | luteolin | AVPR2 | P30518 | × |
| MOL000006 | luteolin | AURKB | Q96GD4 | × |
| MOL000006 | luteolin | CA3 | P07451 | × |
| MOL000006 | luteolin | CA6 | P23280 | × |
| MOL000006 | luteolin | CA14 | Q9ULX7 | × |
| MOL000006 | luteolin | CAMK2B | Q13554 | × |
| MOL000006 | luteolin | ALK | Q9UM73 | × |
| MOL000006 | luteolin | AKT1 | P31749 | × |
| MOL000006 | luteolin | CA5A | P35218 | × |
| MOL000006 | luteolin | BACE1 | P56817 | × |
| MOL000006 | luteolin | AXL | P30530 | × |
| MOL000006 | luteolin | AKR1C2 | P52895 | × |
| MOL000006 | luteolin | AKR1C1 | Q04828 | × |
| MOL000006 | luteolin | AKR1C3 | P42330 | × |
| MOL000006 | luteolin | AKR1C4 | P17516 | × |
| MOL000006 | luteolin | CA13 | Q8N1Q1 | × |
| MOL000006 | luteolin | AKR1A1 | P14550 | × |
| MOL000006 | luteolin | AR | P10275 | × |
| MOL000098 | quercetin | NOX4 | Q9NPH5 | √ |
| MOL000098 | quercetin | XDH | P47989 | √ |
| MOL000098 | quercetin | MAOA | P21397 | √ |
| MOL000098 | quercetin | IGF1R | P08069 | √ |
| MOL000098 | quercetin | FLT3 | P36888 | √ |
| MOL000098 | quercetin | CYP19A1 | P11511 | √ |
| MOL000098 | quercetin | EGFR | P00533 | √ |
| MOL000098 | quercetin | F2 | P00734 | √ |
| MOL000098 | quercetin | PIM1 | P11309 | √ |
| MOL000098 | quercetin | DRD4 | P21917 | √ |
| MOL000098 | quercetin | GLO1 | Q04760 | √ |
| MOL000098 | quercetin | MPO | P05164 | √ |
| MOL000098 | quercetin | PIK3R1 | P27986 | √ |
| MOL000098 | quercetin | DAPK1 | P53355 | √ |
| MOL000098 | quercetin | PYGL | P06737 | √ |
| MOL000098 | quercetin | GSK3B | P49841 | √ |
| MOL000098 | quercetin | SRC | P12931 | √ |
| MOL000098 | quercetin | PTK2 | Q05397 | √ |
| MOL000098 | quercetin | HSD17B2 | P37059 | √ |
| MOL000098 | quercetin | KDR | P35968 | √ |
| MOL000098 | quercetin | MMP13 | P45452 | √ |
| MOL000098 | quercetin | MMP3 | P08254 | √ |
| MOL000098 | quercetin | PLK1 | P53350 | √ |
| MOL000098 | quercetin | CDK1 | P06493 | √ |
| MOL000098 | quercetin | MMP9 | P14780 | √ |
| MOL000098 | quercetin | MMP2 | P08253 | √ |
| MOL000098 | quercetin | PKN1 | Q16512 | √ |
| MOL000098 | quercetin | CSNK2A1 | P68400 | √ |
| MOL000098 | quercetin | MET | P08581 | √ |
| MOL000098 | quercetin | NEK2 | P51955 | √ |
| MOL000098 | quercetin | CXCR1 | P25024 | √ |
| MOL000098 | quercetin | NEK6 | Q9HC98 | √ |
| MOL000098 | quercetin | PLA2G1B | P04054 | √ |
| MOL000098 | quercetin | CYP1B1 | Q16678 | √ |
| MOL000098 | quercetin | NUAK1 | O60285 | √ |
| MOL000098 | quercetin | GPR35 | Q9HC97 | √ |
| MOL000098 | quercetin | MAPT | P10636 | √ |
| MOL000098 | quercetin | KDM4E | B2RXH2 | √ |
| MOL000098 | quercetin | TOP2A | P11388 | √ |
| MOL000098 | quercetin | INSR | P06213 | √ |
| MOL000098 | quercetin | MYLK | Q15746 | √ |
| MOL000098 | quercetin | SYK | P43405 | √ |
| MOL000098 | quercetin | PIK3CG | P48736 | √ |
| MOL000098 | quercetin | PTPRS | Q13332 | √ |
| MOL000098 | quercetin | ESR2 | Q92731 | √ |
| MOL000098 | quercetin | MPG | P29372 | √ |
| MOL000098 | quercetin | SLC22A12 | Q96S37 | √ |
| MOL000098 | quercetin | CDK6 | Q00534 | √ |
| MOL000098 | quercetin | CDK2 | P24941 | √ |
| MOL000098 | quercetin | TYR | P14679 | √ |
| MOL000098 | quercetin | HSD17B1 | P14061 | √ |
| MOL000098 | quercetin | ESRRA | P11474 | √ |
| MOL000098 | quercetin | PARP1 | P09874 | √ |
| MOL000098 | quercetin | TTR | P02766 | √ |
| MOL000098 | quercetin | MMP12 | P39900 | √ |
| MOL000098 | quercetin | CD38 | P28907 | √ |
| MOL000098 | quercetin | TNKS2 | Q9H2K2 | √ |
| MOL000098 | quercetin | TNKS | O95271 | √ |
| MOL000098 | quercetin | TOP1 | P11387 | √ |
| MOL000098 | quercetin | TERT | O14746 | √ |
| MOL000098 | quercetin | CDK5R1 | Q15078 | √ |
| MOL000098 | quercetin | CDK5 | Q00535 | √ |
| MOL000098 | quercetin | CDK1 | P06493 | √ |
| MOL000098 | quercetin | AVPR2 | P30518 | × |
| MOL000098 | quercetin | AKR1B1 | P15121 | × |
| MOL000098 | quercetin | CA2 | P00918 | × |
| MOL000098 | quercetin | ALOX5 | P09917 | × |
| MOL000098 | quercetin | AURKB | Q96GD4 | × |
| MOL000098 | quercetin | ADORA1 | P30542 | × |
| MOL000098 | quercetin | CA7 | P43166 | × |
| MOL000098 | quercetin | ADORA2A | P29274 | × |
| MOL000098 | quercetin | CA1 | P00915 | × |
| MOL000098 | quercetin | CA3 | P07451 | × |
| MOL000098 | quercetin | ALOX15 | P16050 | × |
| MOL000098 | quercetin | ABCC1 | P33527 | × |
| MOL000098 | quercetin | CA6 | P23280 | × |
| MOL000098 | quercetin | CA12 | O43570 | × |
| MOL000098 | quercetin | CA14 | Q9ULX7 | × |
| MOL000098 | quercetin | CA9 | Q16790 | × |
| MOL000098 | quercetin | ALOX12 | P18054 | × |
| MOL000098 | quercetin | CA4 | P22748 | × |
| MOL000098 | quercetin | CAMK2B | Q13554 | × |
| MOL000098 | quercetin | ALK | Q9UM73 | × |
| MOL000098 | quercetin | AKT1 | P31749 | × |
| MOL000098 | quercetin | ABCB1 | P08183 | × |
| MOL000098 | quercetin | CA5A | P35218 | × |
| MOL000098 | quercetin | BACE1 | P56817 | × |
| MOL000098 | quercetin | AXL | P30530 | × |
| MOL000098 | quercetin | ABCG2 | Q9UNQ0 | × |
| MOL000098 | quercetin | AKR1C2 | P52895 | × |
| MOL000098 | quercetin | AKR1C1 | Q04828 | × |
| MOL000098 | quercetin | AKR1C3 | P42330 | × |
| MOL000098 | quercetin | AKR1C4 | P17516 | × |
| MOL000098 | quercetin | CA13 | Q8N1Q1 | × |
| MOL000098 | quercetin | AKR1A1 | P14550 | × |
| MOL000098 | quercetin | ACHE | P22303 | × |
| MOL000098 | quercetin | APEX1 | P27695 | × |
| MOL000098 | quercetin | ARG1 | P05089 | × |
| MOL000098 | quercetin | AHR | P35869 | × |
| MOL000098 | quercetin | APP | P05067 | × |
| MOL000098 | quercetin | AKR1B10 | O60218 | × |
| MOL000098 | quercetin | CCNB3 | Q8WWL7 | × |
| MOL000098 | quercetin | CCNB1 | P14635 | × |
| MOL000098 | quercetin | CCNB2 | O95067 | × |
| MOL000358 | beta-sitosterol | NPC1L1 | Q9UHC9 | √ |
| MOL000358 | beta-sitosterol | NR1H3 | Q13133 | √ |
| MOL000358 | beta-sitosterol | RORC | P51449 | √ |
| MOL000358 | beta-sitosterol | SHBG | P04278 | √ |
| MOL000358 | beta-sitosterol | HMGCR | P04035 | √ |
| MOL000358 | beta-sitosterol | CYP17A1 | P05093 | √ |
| MOL000358 | beta-sitosterol | SREBF2 | Q12772 | √ |
| MOL000358 | beta-sitosterol | CYP19A1 | P11511 | √ |
| MOL000358 | beta-sitosterol | CYP51A1 | Q16850 | √ |
| MOL000358 | beta-sitosterol | RORA | P35398 | √ |
| MOL000358 | beta-sitosterol | ESR1 | P03372 | √ |
| MOL000358 | beta-sitosterol | ESR2 | Q92731 | √ |
| MOL000358 | beta-sitosterol | PTPN1 | P18031 | √ |
| MOL000358 | beta-sitosterol | CYP2C19 | P33261 | √ |
| MOL000358 | beta-sitosterol | SERPINA6 | P08185 | √ |
| MOL000358 | beta-sitosterol | G6PD | P11413 | √ |
| MOL000358 | beta-sitosterol | SLC6A2 | P23975 | √ |
| MOL000358 | beta-sitosterol | SLC6A4 | P31645 | √ |
| MOL000358 | beta-sitosterol | CHRM2 | P08172 | √ |
| MOL000358 | beta-sitosterol | NR1I3 | Q14994 | √ |
| MOL000358 | beta-sitosterol | NR1H2 | P55055 | √ |
| MOL000358 | beta-sitosterol | VDR | P11473 | √ |
| MOL000358 | beta-sitosterol | PTGER1 | P34995 | √ |
| MOL000358 | beta-sitosterol | PTGER2 | P43116 | √ |
| MOL000358 | beta-sitosterol | TBXAS1 | P24557 | √ |
| MOL000358 | beta-sitosterol | PTGES | O14684 | √ |
| MOL000358 | beta-sitosterol | DHCR7 | Q9UBM7 | √ |
| MOL000358 | beta-sitosterol | PPARD | Q03181 | √ |
| MOL000358 | beta-sitosterol | SQLE | Q14534 | √ |
| MOL000358 | beta-sitosterol | GLRA1 | P23415 | √ |
| MOL000358 | beta-sitosterol | HSD11B1 | P28845 | √ |
| MOL000358 | beta-sitosterol | PTPN6 | P29350 | √ |
| MOL000358 | beta-sitosterol | PTPN2 | P17706 | √ |
| MOL000358 | beta-sitosterol | FDFT1 | P37268 | √ |
| MOL000358 | beta-sitosterol | CES2 | O00748 | √ |
| MOL000358 | beta-sitosterol | NOS2 | P35228 | √ |
| MOL000358 | beta-sitosterol | PPARG | P37231 | √ |
| MOL000358 | beta-sitosterol | UGT2B7 | P16662 | √ |
| MOL000358 | beta-sitosterol | NR3C1 | P04150 | √ |
| MOL000358 | beta-sitosterol | POLB | P06746 | √ |
| MOL000358 | beta-sitosterol | PREP | P48147 | √ |
| MOL000358 | beta-sitosterol | SIGMAR1 | Q99720 | √ |
| MOL000358 | beta-sitosterol | PTGER4 | P35408 | √ |
| MOL000358 | beta-sitosterol | IDO1 | P14902 | √ |
| MOL000358 | beta-sitosterol | DNM1 | Q05193 | √ |
| MOL000358 | beta-sitosterol | ICMT | O60725 | √ |
| MOL000358 | beta-sitosterol | SMO | Q99835 | √ |
| MOL000358 | beta-sitosterol | PTGIR | P43119 | √ |
| MOL000358 | beta-sitosterol | FABP4 | P15090 | √ |
| MOL000358 | beta-sitosterol | TERT | O14746 | √ |
| MOL000358 | beta-sitosterol | FABP3 | P05413 | √ |
| MOL000358 | beta-sitosterol | FABP5 | Q01469 | √ |
| MOL000358 | beta-sitosterol | FABP1 | P07148 | √ |
| MOL000358 | beta-sitosterol | MDM2 | Q00987 | √ |
| MOL000358 | beta-sitosterol | SLC6A3 | Q01959 | √ |
| MOL000358 | beta-sitosterol | MAPK3 | P27361 | √ |
| MOL000358 | beta-sitosterol | PRKCH | P24723 | √ |
| MOL000358 | beta-sitosterol | PTPN11 | Q06124 | √ |
| MOL000358 | beta-sitosterol | S1PR3 | Q99500 | √ |
| MOL000358 | beta-sitosterol | S1PR1 | P21453 | √ |
| MOL000358 | beta-sitosterol | CXCR3 | P49682 | √ |
| MOL000358 | beta-sitosterol | PTPRF | P10586 | √ |
| MOL000358 | beta-sitosterol | PLA2G1B | P04054 | √ |
| MOL000358 | beta-sitosterol | PYGL | P06737 | √ |
| MOL000358 | beta-sitosterol | PDE4A | P27815 | √ |
| MOL000358 | beta-sitosterol | PDE4B | Q07343 | √ |
| MOL000358 | beta-sitosterol | PDE4C | Q08493 | √ |
| MOL000358 | beta-sitosterol | SHH | Q15465 | √ |
| MOL000358 | beta-sitosterol | METAP1 | P53582 | √ |
| MOL000358 | beta-sitosterol | HSD11B2 | P80365 | √ |
| MOL000358 | beta-sitosterol | HCRTR2 | O43614 | √ |
| MOL000358 | beta-sitosterol | HCRTR1 | O43613 | √ |
| MOL000358 | beta-sitosterol | GC | P02774 | √ |
| MOL000358 | beta-sitosterol | PPARA | Q07869 | √ |
| MOL000358 | beta-sitosterol | TOP2A | P11388 | √ |
| MOL000358 | beta-sitosterol | F2 | P00734 | √ |
| MOL000358 | beta-sitosterol | F10 | P00742 | √ |
| MOL000358 | beta-sitosterol | TNF | P01375 | √ |
| MOL000358 | beta-sitosterol | PTGFR | P43088 | √ |
| MOL000358 | beta-sitosterol | PTGER3 | P43115 | √ |
| MOL000358 | beta-sitosterol | PTGDR | Q13258 | √ |
| MOL000358 | beta-sitosterol | TOP1 | P11387 | √ |
| MOL000358 | beta-sitosterol | NR3C2 | P08235 | √ |
| MOL000358 | beta-sitosterol | HIF1A | Q16665 | √ |
| MOL000358 | beta-sitosterol | MAPK8 | P45983 | √ |
| MOL000358 | beta-sitosterol | MAST3 | O60307 | √ |
| MOL000358 | beta-sitosterol | DRD2 | P14416 | √ |
| MOL000358 | beta-sitosterol | SRD5A2 | P31213 | √ |
| MOL000358 | beta-sitosterol | MGLL | Q99685 | √ |
| MOL000358 | beta-sitosterol | DGAT1 | O75907 | √ |
| MOL000358 | beta-sitosterol | DHCR7 | Q9UBM7 | √ |
| MOL000358 | beta-sitosterol | EBP | Q15125 | √ |
| MOL000358 | beta-sitosterol | ITGAL | P20701 | √ |
| MOL000358 | beta-sitosterol | ICAM1 | P05362 | √ |
| MOL000358 | beta-sitosterol | ITGB2 | P05107 | √ |
| MOL000358 | beta-sitosterol | AR | P10275 | × |
| MOL000358 | beta-sitosterol | ACHE | P22303 | × |
| MOL000358 | beta-sitosterol | BCHE | P06276 | × |
| MOL000358 | beta-sitosterol | ATP12A | P54707 | × |
| MOL000358 | beta-sitosterol | ADORA3 | P0DMS8 | × |
| MOL000358 | beta-sitosterol | AKR1B10 | O60218 | × |
| MOL000358 | beta-sitosterol | ACP1 | P24666 | × |
| MOL000358 | beta-sitosterol | CCR1 | P32246 | × |
| MOL000422 | kaempferol | NOX4 | Q9NPH5 | √ |
| MOL000422 | kaempferol | XDH | P47989 | √ |
| MOL000422 | kaempferol | TYR | P14679 | √ |
| MOL000422 | kaempferol | FLT3 | P36888 | √ |
| MOL000422 | kaempferol | HSD17B2 | P37059 | √ |
| MOL000422 | kaempferol | HSD17B1 | P14061 | √ |
| MOL000422 | kaempferol | ESRRA | P11474 | √ |
| MOL000422 | kaempferol | CYP1B1 | Q16678 | √ |
| MOL000422 | kaempferol | MAOA | P21397 | √ |
| MOL000422 | kaempferol | GLO1 | Q04760 | √ |
| MOL000422 | kaempferol | SYK | P43405 | √ |
| MOL000422 | kaempferol | GSK3B | P49841 | √ |
| MOL000422 | kaempferol | MMP9 | P14780 | √ |
| MOL000422 | kaempferol | MMP2 | P08253 | √ |
| MOL000422 | kaempferol | PTPRS | Q13332 | √ |
| MOL000422 | kaempferol | GPR35 | Q9HC97 | √ |
| MOL000422 | kaempferol | ESR2 | Q92731 | √ |
| MOL000422 | kaempferol | DAPK1 | P53355 | √ |
| MOL000422 | kaempferol | MPG | P29372 | √ |
| MOL000422 | kaempferol | SLC22A12 | Q96S37 | √ |
| MOL000422 | kaempferol | TTR | P02766 | √ |
| MOL000422 | kaempferol | TNKS2 | Q9H2K2 | √ |
| MOL000422 | kaempferol | TNKS | O95271 | √ |
| MOL000422 | kaempferol | CDK6 | Q00534 | √ |
| MOL000422 | kaempferol | CDK2 | P24941 | √ |
| MOL000422 | kaempferol | CYP19A1 | P11511 | √ |
| MOL000422 | kaempferol | CSNK2A1 | P68400 | √ |
| MOL000422 | kaempferol | EGFR | P00533 | √ |
| MOL000422 | kaempferol | IGF1R | P08069 | √ |
| MOL000422 | kaempferol | F2 | P00734 | √ |
| MOL000422 | kaempferol | PIM1 | P11309 | √ |
| MOL000422 | kaempferol | DRD4 | P21917 | √ |
| MOL000422 | kaempferol | MPO | P05164 | √ |
| MOL000422 | kaempferol | PIK3R1 | P27986 | √ |
| MOL000422 | kaempferol | PYGL | P06737 | √ |
| MOL000422 | kaempferol | SRC | P12931 | √ |
| MOL000422 | kaempferol | PTK2 | Q05397 | √ |
| MOL000422 | kaempferol | KDR | P35968 | √ |
| MOL000422 | kaempferol | MMP13 | P45452 | √ |
| MOL000422 | kaempferol | MMP3 | P08254 | √ |
| MOL000422 | kaempferol | PLK1 | P53350 | √ |
| MOL000422 | kaempferol | CDK1 | P06493 | √ |
| MOL000422 | kaempferol | PKN1 | Q16512 | √ |
| MOL000422 | kaempferol | MET | P08581 | √ |
| MOL000422 | kaempferol | NEK2 | P51955 | √ |
| MOL000422 | kaempferol | CXCR1 | P25024 | √ |
| MOL000422 | kaempferol | NEK6 | Q9HC98 | √ |
| MOL000422 | kaempferol | PLA2G1B | P04054 | √ |
| MOL000422 | kaempferol | NUAK1 | O60285 | √ |
| MOL000422 | kaempferol | PARP1 | P09874 | √ |
| MOL000422 | kaempferol | MMP12 | P39900 | √ |
| MOL000422 | kaempferol | CD38 | P28907 | √ |
| MOL000422 | kaempferol | TOP1 | P11387 | √ |
| MOL000422 | kaempferol | ESR1 | P03372 | √ |
| MOL000422 | kaempferol | PTGS2 | P35354 | √ |
| MOL000422 | kaempferol | CFTR | P13569 | √ |
| MOL000422 | kaempferol | PFKFB3 | Q16875 | √ |
| MOL000422 | kaempferol | GRK6 | P43250 | √ |
| MOL000422 | kaempferol | TERT | O14746 | √ |
| MOL000422 | kaempferol | MAPT | P10636 | √ |
| MOL000422 | kaempferol | CDK5R1 | Q15078 | √ |
| MOL000422 | kaempferol | CDK5 | Q00535 | √ |
| MOL000422 | kaempferol | CDK1 | P06493 | √ |
| MOL000422 | kaempferol | AKR1B1 | P15121 | × |
| MOL000422 | kaempferol | CA2 | P00918 | × |
| MOL000422 | kaempferol | ALOX5 | P09917 | × |
| MOL000422 | kaempferol | CA7 | P43166 | × |
| MOL000422 | kaempferol | ABCC1 | P33527 | × |
| MOL000422 | kaempferol | AHR | P35869 | × |
| MOL000422 | kaempferol | CA12 | O43570 | × |
| MOL000422 | kaempferol | ABCB1 | P08183 | × |
| MOL000422 | kaempferol | ABCG2 | Q9UNQ0 | × |
| MOL000422 | kaempferol | ADORA1 | P30542 | × |
| MOL000422 | kaempferol | CA4 | P22748 | × |
| MOL000422 | kaempferol | ACHE | P22303 | × |
| MOL000422 | kaempferol | ALOX15 | P16050 | × |
| MOL000422 | kaempferol | ALOX12 | P18054 | × |
| MOL000422 | kaempferol | ADORA2A | P29274 | × |
| MOL000422 | kaempferol | ARG1 | P05089 | × |
| MOL000422 | kaempferol | AKR1B10 | O60218 | × |
| MOL000422 | kaempferol | AVPR2 | P30518 | × |
| MOL000422 | kaempferol | AURKB | Q96GD4 | × |
| MOL000422 | kaempferol | CA1 | P00915 | × |
| MOL000422 | kaempferol | CA3 | P07451 | × |
| MOL000422 | kaempferol | CA6 | P23280 | × |
| MOL000422 | kaempferol | CA14 | Q9ULX7 | × |
| MOL000422 | kaempferol | CA9 | Q16790 | × |
| MOL000422 | kaempferol | CAMK2B | Q13554 | × |
| MOL000422 | kaempferol | ALK | Q9UM73 | × |
| MOL000422 | kaempferol | AKT1 | P31749 | × |
| MOL000422 | kaempferol | CA5A | P35218 | × |
| MOL000422 | kaempferol | BACE1 | P56817 | × |
| MOL000422 | kaempferol | AXL | P30530 | × |
| MOL000422 | kaempferol | AKR1C2 | P52895 | × |
| MOL000422 | kaempferol | AKR1C1 | Q04828 | × |
| MOL000422 | kaempferol | AKR1C3 | P42330 | × |
| MOL000422 | kaempferol | AKR1C4 | P17516 | × |
| MOL000422 | kaempferol | CA13 | Q8N1Q1 | × |
| MOL000422 | kaempferol | AKR1A1 | P14550 | × |
| MOL000422 | kaempferol | APP | P05067 | × |
| MOL000422 | kaempferol | AMY1A | P04745 | × |
| MOL000422 | kaempferol | CCNB3 | Q8WWL7 | × |
| MOL000422 | kaempferol | CCNB1 | P14635 | × |
| MOL000422 | kaempferol | CCNB2 | O95067 | × |
| MOL000449 | Stigmasterol | NPC1L1 | Q9UHC9 | √ |
| MOL000449 | Stigmasterol | NR1H3 | Q13133 | √ |
| MOL000449 | Stigmasterol | RORC | P51449 | √ |
| MOL000449 | Stigmasterol | ESR1 | P03372 | √ |
| MOL000449 | Stigmasterol | ESR2 | Q92731 | √ |
| MOL000449 | Stigmasterol | SHBG | P04278 | √ |
| MOL000449 | Stigmasterol | SREBF2 | Q12772 | √ |
| MOL000449 | Stigmasterol | HMGCR | P04035 | √ |
| MOL000449 | Stigmasterol | CYP19A1 | P11511 | √ |
| MOL000449 | Stigmasterol | CYP17A1 | P05093 | √ |
| MOL000449 | Stigmasterol | CYP51A1 | Q16850 | √ |
| MOL000449 | Stigmasterol | RORA | P35398 | √ |
| MOL000449 | Stigmasterol | CYP2C19 | P33261 | √ |
| MOL000449 | Stigmasterol | PTPN1 | P18031 | √ |
| MOL000449 | Stigmasterol | SERPINA6 | P08185 | √ |
| MOL000449 | Stigmasterol | G6PD | P11413 | √ |
| MOL000449 | Stigmasterol | SLC6A4 | P31645 | √ |
| MOL000449 | Stigmasterol | NR1I3 | Q14994 | √ |
| MOL000449 | Stigmasterol | CHRM2 | P08172 | √ |
| MOL000449 | Stigmasterol | SLC6A2 | P23975 | √ |
| MOL000449 | Stigmasterol | NR1H2 | P55055 | √ |
| MOL000449 | Stigmasterol | PTGER1 | P34995 | √ |
| MOL000449 | Stigmasterol | PTGER2 | P43116 | √ |
| MOL000449 | Stigmasterol | TBXAS1 | P24557 | √ |
| MOL000449 | Stigmasterol | PTGES | O14684 | √ |
| MOL000449 | Stigmasterol | PPARA | Q07869 | √ |
| MOL000449 | Stigmasterol | PPARD | Q03181 | √ |
| MOL000449 | Stigmasterol | SQLE | Q14534 | √ |
| MOL000449 | Stigmasterol | VDR | P11473 | √ |
| MOL000449 | Stigmasterol | DHCR7 | Q9UBM7 | √ |
| MOL000449 | Stigmasterol | PTPN6 | P29350 | √ |
| MOL000449 | Stigmasterol | PTPN2 | P17706 | √ |
| MOL000449 | Stigmasterol | FDFT1 | P37268 | √ |
| MOL000449 | Stigmasterol | HSD11B1 | P28845 | √ |
| MOL000449 | Stigmasterol | NOS2 | P35228 | √ |
| MOL000449 | Stigmasterol | PPARG | P37231 | √ |
| MOL000449 | Stigmasterol | UGT2B7 | P16662 | √ |
| MOL000449 | Stigmasterol | GLRA1 | P23415 | √ |
| MOL000449 | Stigmasterol | POLB | P06746 | √ |
| MOL000449 | Stigmasterol | AR | P10275 | × |
| MOL000449 | Stigmasterol | ACHE | P22303 | × |
| MOL000449 | Stigmasterol | BCHE | P06276 | × |
| MOL001494 | Mandenol | FAAH | O00519 | √ |
| MOL001494 | Mandenol | CNR1 | P21554 | √ |
| MOL001494 | Mandenol | CNR2 | P34972 | √ |
| MOL001494 | Mandenol | CYP17A1 | P05093 | √ |
| MOL001494 | Mandenol | CYP19A1 | P11511 | √ |
| MOL001494 | Mandenol | CES2 | O00748 | √ |
| MOL001494 | Mandenol | HSD11B1 | P28845 | √ |
| MOL001494 | Mandenol | FABP4 | P15090 | √ |
| MOL001494 | Mandenol | FABP3 | P05413 | √ |
| MOL001494 | Mandenol | PTGES | O14684 | √ |
| MOL001494 | Mandenol | PPARG | P37231 | √ |
| MOL001494 | Mandenol | PTGS1 | P23219 | √ |
| MOL001494 | Mandenol | PTPN1 | P18031 | √ |
| MOL001494 | Mandenol | PTGS2 | P35354 | √ |
| MOL001494 | Mandenol | HSD17B2 | P37059 | √ |
| MOL001494 | Mandenol | NR3C1 | P04150 | √ |
| MOL001494 | Mandenol | PGR | P06401 | √ |
| MOL001494 | Mandenol | TNKS | O95271 | √ |
| MOL001494 | Mandenol | PRKCQ | Q04759 | √ |
| MOL001494 | Mandenol | HMGCR | P04035 | √ |
| MOL001494 | Mandenol | PRKCD | Q05655 | √ |
| MOL001494 | Mandenol | LIMK2 | P53671 | √ |
| MOL001494 | Mandenol | CDC7 | O00311 | √ |
| MOL001494 | Mandenol | NR1H3 | Q13133 | √ |
| MOL001494 | Mandenol | FFAR1 | O14842 | √ |
| MOL001494 | Mandenol | PFKFB3 | Q16875 | √ |
| MOL001494 | Mandenol | PPARA | Q07869 | √ |
| MOL001494 | Mandenol | HRH3 | Q9Y5N1 | √ |
| MOL001494 | Mandenol | HRH4 | Q9H3N8 | √ |
| MOL001494 | Mandenol | FABP5 | Q01469 | √ |
| MOL001494 | Mandenol | FABP1 | P07148 | √ |
| MOL001494 | Mandenol | F2R | P25116 | √ |
| MOL001494 | Mandenol | NR3C2 | P08235 | √ |
| MOL001494 | Mandenol | CDK1 | P06493 | √ |
| MOL001494 | Mandenol | PPARD | Q03181 | √ |
| MOL001494 | Mandenol | GRIN2B | Q13224 | √ |
| MOL001494 | Mandenol | RARG | P13631 | √ |
| MOL001494 | Mandenol | RARB | P10826 | √ |
| MOL001494 | Mandenol | RARA | P10276 | √ |
| MOL001494 | Mandenol | CTSK | P43235 | √ |
| MOL001494 | Mandenol | RXRA | P19793 | √ |
| MOL001494 | Mandenol | RXRG | P48443 | √ |
| MOL001494 | Mandenol | NPY5R | Q15761 | √ |
| MOL001494 | Mandenol | PRKCA | P17252 | √ |
| MOL001494 | Mandenol | NPY2R | P49146 | √ |
| MOL001494 | Mandenol | SLC6A9 | P48067 | √ |
| MOL001494 | Mandenol | FLT1 | P17948 | √ |
| MOL001494 | Mandenol | PDGFRB | P09619 | √ |
| MOL001494 | Mandenol | KIT | P10721 | √ |
| MOL001494 | Mandenol | MTNR1A | P48039 | √ |
| MOL001494 | Mandenol | MTNR1B | P49286 | √ |
| MOL001494 | Mandenol | KDR | P35968 | √ |
| MOL001494 | Mandenol | GCK | P35557 | √ |
| MOL001494 | Mandenol | QPCT | Q16769 | √ |
| MOL001494 | Mandenol | SCD | O00767 | √ |
| MOL001494 | Mandenol | OXTR | P30559 | √ |
| MOL001494 | Mandenol | PTGIR | P43119 | √ |
| MOL001494 | Mandenol | JAK1 | P23458 | √ |
| MOL001494 | Mandenol | EPHX1 | P07099 | √ |
| MOL001494 | Mandenol | SRC | P12931 | √ |
| MOL001494 | Mandenol | PARP2 | Q9UGN5 | √ |
| MOL001494 | Mandenol | JAK2 | O60674 | √ |
| MOL001494 | Mandenol | PDE10A | Q9Y233 | √ |
| MOL001494 | Mandenol | PTAFR | P25105 | √ |
| MOL001494 | Mandenol | POLB | P06746 | √ |
| MOL001494 | Mandenol | MGAT2 | Q10469 | √ |
| MOL001494 | Mandenol | KIF11 | P52732 | √ |
| MOL001494 | Mandenol | HSD11B2 | P80365 | √ |
| MOL001494 | Mandenol | RBP4 | P02753 | √ |
| MOL001494 | Mandenol | TBXA2R | P21731 | √ |
| MOL001494 | Mandenol | PDE6D | O43924 | √ |
| MOL001494 | Mandenol | IMPDH2 | P12268 | √ |
| MOL001494 | Mandenol | ITK | Q08881 | √ |
| MOL001494 | Mandenol | SLC6A15 | Q9H2J7 | √ |
| MOL001494 | Mandenol | CETP | P11597 | √ |
| MOL001494 | Mandenol | MAP3K5 | Q99683 | √ |
| MOL001494 | Mandenol | MAPK1 | P28482 | √ |
| MOL001494 | Mandenol | PTPN6 | P29350 | √ |
| MOL001494 | Mandenol | YES1 | P07947 | √ |
| MOL001494 | Mandenol | PSEN2 | P49810 | √ |
| MOL001494 | Mandenol | PSENEN | Q9NZ42 | √ |
| MOL001494 | Mandenol | NCSTN | Q92542 | √ |
| MOL001494 | Mandenol | PSEN1 | P49768 | √ |
| MOL001494 | Mandenol | GABRB3 | P28472 | √ |
| MOL001494 | Mandenol | GABRA3 | P34903 | √ |
| MOL001494 | Mandenol | GABRG2 | P18507 | √ |
| MOL001494 | Mandenol | GABRA1 | P14867 | √ |
| MOL001494 | Mandenol | GABRA2 | P47869 | √ |
| MOL001494 | Mandenol | GABRA5 | P31644 | √ |
| MOL001494 | Mandenol | FNTA | P49354 | √ |
| MOL001494 | Mandenol | FNTB | P49356 | √ |
| MOL001494 | Mandenol | AR | P10275 | × |
| MOL001494 | Mandenol | C5AR1 | P21730 | × |
| MOL001494 | Mandenol | AKR1C3 | P42330 | × |
| MOL001494 | Mandenol | AKR1C2 | P52895 | × |
| MOL001494 | Mandenol | AKR1C1 | Q04828 | × |
| MOL001494 | Mandenol | ALOX5AP | P20292 | × |
| MOL001494 | Mandenol | ALOX5 | P09917 | × |
| MOL001494 | Mandenol | ASAH1 | Q13510 | × |
| MOL001494 | Mandenol | ABL1 | P00519 | × |
| MOL001494 | Mandenol | ACACA | Q13085 | × |
| MOL001494 | Mandenol | ACACB | O00763 | × |
| MOL001494 | Mandenol | AOC3 | Q16853 | × |
| MOL001494 | Mandenol | BRD4 | O60885 | × |
| MOL001494 | Mandenol | BRD2 | P25440 | × |
| MOL001494 | Mandenol | BRD3 | Q15059 | × |
| MOL001494 | Mandenol | APH1A | Q96BI3 | × |
| MOL001494 | Mandenol | APH1B | Q8WW43 | × |
| MOL001495 | Ethyl linolenate | FAAH | O00519 | √ |
| MOL001495 | Ethyl linolenate | CNR1 | P21554 | √ |
| MOL001495 | Ethyl linolenate | CNR2 | P34972 | √ |
| MOL001495 | Ethyl linolenate | PTGES | O14684 | √ |
| MOL001495 | Ethyl linolenate | CYP19A1 | P11511 | √ |
| MOL001495 | Ethyl linolenate | FABP4 | P15090 | √ |
| MOL001495 | Ethyl linolenate | FABP3 | P05413 | √ |
| MOL001495 | Ethyl linolenate | FFAR1 | O14842 | √ |
| MOL001495 | Ethyl linolenate | PTPN1 | P18031 | √ |
| MOL001495 | Ethyl linolenate | CYP17A1 | P05093 | √ |
| MOL001495 | Ethyl linolenate | HSD11B1 | P28845 | √ |
| MOL001495 | Ethyl linolenate | PTGS1 | P23219 | √ |
| MOL001495 | Ethyl linolenate | CES2 | O00748 | √ |
| MOL001495 | Ethyl linolenate | PPARG | P37231 | √ |
| MOL001495 | Ethyl linolenate | PPARD | Q03181 | √ |
| MOL001495 | Ethyl linolenate | HMGCR | P04035 | √ |
| MOL001495 | Ethyl linolenate | NR1H3 | Q13133 | √ |
| MOL001495 | Ethyl linolenate | NPY5R | Q15761 | √ |
| MOL001495 | Ethyl linolenate | PTGS2 | P35354 | √ |
| MOL001495 | Ethyl linolenate | F2R | P25116 | √ |
| MOL001495 | Ethyl linolenate | SHBG | P04278 | √ |
| MOL001495 | Ethyl linolenate | PTPN2 | P17706 | √ |
| MOL001495 | Ethyl linolenate | IDH1 | O75874 | √ |
| MOL001495 | Ethyl linolenate | PDE10A | Q9Y233 | √ |
| MOL001495 | Ethyl linolenate | FABP5 | Q01469 | √ |
| MOL001495 | Ethyl linolenate | FABP1 | P07148 | √ |
| MOL001495 | Ethyl linolenate | NR3C2 | P08235 | √ |
| MOL001495 | Ethyl linolenate | NR3C1 | P04150 | √ |
| MOL001495 | Ethyl linolenate | PGR | P06401 | √ |
| MOL001495 | Ethyl linolenate | HSD17B2 | P37059 | √ |
| MOL001495 | Ethyl linolenate | CYP26A1 | O43174 | √ |
| MOL001495 | Ethyl linolenate | PPARA | Q07869 | √ |
| MOL001495 | Ethyl linolenate | HRH3 | Q9Y5N1 | √ |
| MOL001495 | Ethyl linolenate | HRH4 | Q9H3N8 | √ |
| MOL001495 | Ethyl linolenate | TBXA2R | P21731 | √ |
| MOL001495 | Ethyl linolenate | PTGER2 | P43116 | √ |
| MOL001495 | Ethyl linolenate | PTGIR | P43119 | √ |
| MOL001495 | Ethyl linolenate | HSD11B2 | P80365 | √ |
| MOL001495 | Ethyl linolenate | CYP26B1 | Q9NR63 | √ |
| MOL001495 | Ethyl linolenate | SMO | Q99835 | √ |
| MOL001495 | Ethyl linolenate | TGM2 | P21980 | √ |
| MOL001495 | Ethyl linolenate | TGM1 | P22735 | √ |
| MOL001495 | Ethyl linolenate | NPY1R | P25929 | √ |
| MOL001495 | Ethyl linolenate | TNKS | O95271 | √ |
| MOL001495 | Ethyl linolenate | GRIN2B | Q13224 | √ |
| MOL001495 | Ethyl linolenate | FLT1 | P17948 | √ |
| MOL001495 | Ethyl linolenate | RAF1 | P04049 | √ |
| MOL001495 | Ethyl linolenate | PDGFRB | P09619 | √ |
| MOL001495 | Ethyl linolenate | KIT | P10721 | √ |
| MOL001495 | Ethyl linolenate | FLT4 | P35916 | √ |
| MOL001495 | Ethyl linolenate | SRC | P12931 | √ |
| MOL001495 | Ethyl linolenate | KDR | P35968 | √ |
| MOL001495 | Ethyl linolenate | PRKCD | Q05655 | √ |
| MOL001495 | Ethyl linolenate | PRKCQ | Q04759 | √ |
| MOL001495 | Ethyl linolenate | QPCT | Q16769 | √ |
| MOL001495 | Ethyl linolenate | PLD1 | Q13393 | √ |
| MOL001495 | Ethyl linolenate | PLD2 | O14939 | √ |
| MOL001495 | Ethyl linolenate | GRIA2 | P42262 | √ |
| MOL001495 | Ethyl linolenate | EPHX1 | P07099 | √ |
| MOL001495 | Ethyl linolenate | PFKFB3 | Q16875 | √ |
| MOL001495 | Ethyl linolenate | NLRP3 | Q96P20 | √ |
| MOL001495 | Ethyl linolenate | MAOB | P27338 | √ |
| MOL001495 | Ethyl linolenate | NAAA | Q02083 | √ |
| MOL001495 | Ethyl linolenate | CRHR1 | P34998 | √ |
| MOL001495 | Ethyl linolenate | OXTR | P30559 | √ |
| MOL001495 | Ethyl linolenate | GRM5 | P41594 | √ |
| MOL001495 | Ethyl linolenate | PARP1 | P09874 | √ |
| MOL001495 | Ethyl linolenate | TNKS2 | Q9H2K2 | √ |
| MOL001495 | Ethyl linolenate | KCNK9 | Q9NPC2 | √ |
| MOL001495 | Ethyl linolenate | MAPK14 | Q16539 | √ |
| MOL001495 | Ethyl linolenate | CDK2 | P24941 | √ |
| MOL001495 | Ethyl linolenate | CDK7 | P50613 | √ |
| MOL001495 | Ethyl linolenate | CDK1 | P06493 | √ |
| MOL001495 | Ethyl linolenate | CDK9 | P50750 | √ |
| MOL001495 | Ethyl linolenate | CDK4 | P11802 | √ |
| MOL001495 | Ethyl linolenate | CDK5 | Q00535 | √ |
| MOL001495 | Ethyl linolenate | GNRHR | P30968 | √ |
| MOL001495 | Ethyl linolenate | SSTR2 | P30874 | √ |
| MOL001495 | Ethyl linolenate | SSTR4 | P31391 | √ |
| MOL001495 | Ethyl linolenate | CTSS | P25774 | √ |
| MOL001495 | Ethyl linolenate | CTSL | P07711 | √ |
| MOL001495 | Ethyl linolenate | CDC7 | O00311 | √ |
| MOL001495 | Ethyl linolenate | CES1 | P23141 | √ |
| MOL001495 | Ethyl linolenate | NAMPT | P43490 | √ |
| MOL001495 | Ethyl linolenate | PSEN2 | P49810 | √ |
| MOL001495 | Ethyl linolenate | PSENEN | Q9NZ42 | √ |
| MOL001495 | Ethyl linolenate | NCSTN | Q92542 | √ |
| MOL001495 | Ethyl linolenate | PSEN1 | P49768 | √ |
| MOL001495 | Ethyl linolenate | FNTA | P49354 | √ |
| MOL001495 | Ethyl linolenate | FNTB | P49356 | √ |
| MOL001495 | Ethyl linolenate | CDK1 | P06493 | √ |
| MOL001495 | Ethyl linolenate | INCENP | Q9NQS7 | √ |
| MOL001495 | Ethyl linolenate | CDK2 | P24941 | √ |
| MOL001495 | Ethyl linolenate | AR | P10275 | × |
| MOL001495 | Ethyl linolenate | AKR1C2 | P52895 | × |
| MOL001495 | Ethyl linolenate | AKR1C1 | Q04828 | × |
| MOL001495 | Ethyl linolenate | ALOX5AP | P20292 | × |
| MOL001495 | Ethyl linolenate | ADORA2A | P29274 | × |
| MOL001495 | Ethyl linolenate | AVPR2 | P30518 | × |
| MOL001495 | Ethyl linolenate | ACACB | O00763 | × |
| MOL001495 | Ethyl linolenate | AVPR1A | P37288 | × |
| MOL001495 | Ethyl linolenate | ABL1 | P00519 | × |
| MOL001495 | Ethyl linolenate | AURKA | O14965 | × |
| MOL001495 | Ethyl linolenate | BTK | Q06187 | × |
| MOL001495 | Ethyl linolenate | APH1A | Q96BI3 | × |
| MOL001495 | Ethyl linolenate | APH1B | Q8WW43 | × |
| MOL001495 | Ethyl linolenate | CCNB3 | Q8WWL7 | × |
| MOL001495 | Ethyl linolenate | CCNB1 | P14635 | × |
| MOL001495 | Ethyl linolenate | CCNB2 | O95067 | × |
| MOL001495 | Ethyl linolenate | AURKB | Q96GD4 | × |
| MOL001495 | Ethyl linolenate | CCNE1 | P24864 | × |
| MOL002773 | beta-carotene | RBP4 | P02753 | √ |
| MOL002914 | Eriodyctiol (flavanone) | CYP19A1 | P11511 | √ |
| MOL002914 | Eriodyctiol (flavanone) | CYP1B1 | Q16678 | √ |
| MOL002914 | Eriodyctiol (flavanone) | ESR1 | P03372 | √ |
| MOL002914 | Eriodyctiol (flavanone) | ESR2 | Q92731 | √ |
| MOL002914 | Eriodyctiol (flavanone) | HSD17B1 | P14061 | √ |
| MOL002914 | Eriodyctiol (flavanone) | SHBG | P04278 | √ |
| MOL002914 | Eriodyctiol (flavanone) | PTGS1 | P23219 | √ |
| MOL002914 | Eriodyctiol (flavanone) | TAS2R31 | P59538 | √ |
| MOL002914 | Eriodyctiol (flavanone) | MAOB | P27338 | √ |
| MOL002914 | Eriodyctiol (flavanone) | MMP13 | P45452 | √ |
| MOL002914 | Eriodyctiol (flavanone) | MMP12 | P39900 | √ |
| MOL002914 | Eriodyctiol (flavanone) | PLA2G1B | P04054 | √ |
| MOL002914 | Eriodyctiol (flavanone) | KLK1 | P06870 | √ |
| MOL002914 | Eriodyctiol (flavanone) | KLK2 | P20151 | √ |
| MOL002914 | Eriodyctiol (flavanone) | GRM5 | P41594 | √ |
| MOL002914 | Eriodyctiol (flavanone) | CES1 | P23141 | √ |
| MOL002914 | Eriodyctiol (flavanone) | PPARG | P37231 | √ |
| MOL002914 | Eriodyctiol (flavanone) | CES2 | O00748 | √ |
| MOL002914 | Eriodyctiol (flavanone) | SLC5A2 | P31639 | √ |
| MOL002914 | Eriodyctiol (flavanone) | MAOA | P21397 | √ |
| MOL002914 | Eriodyctiol (flavanone) | POLB | P06746 | √ |
| MOL002914 | Eriodyctiol (flavanone) | MAPT | P10636 | √ |
| MOL002914 | Eriodyctiol (flavanone) | PLA2G2A | P14555 | √ |
| MOL002914 | Eriodyctiol (flavanone) | PLA2G5 | P39877 | √ |
| MOL002914 | Eriodyctiol (flavanone) | PLA2G10 | O15496 | √ |
| MOL002914 | Eriodyctiol (flavanone) | IGFBP3 | P17936 | √ |
| MOL002914 | Eriodyctiol (flavanone) | EDNRA | P25101 | √ |
| MOL002914 | Eriodyctiol (flavanone) | SRC | P12931 | √ |
| MOL002914 | Eriodyctiol (flavanone) | SNCA | P37840 | √ |
| MOL002914 | Eriodyctiol (flavanone) | DNMT1 | P26358 | √ |
| MOL002914 | Eriodyctiol (flavanone) | KCNH2 | Q12809 | √ |
| MOL002914 | Eriodyctiol (flavanone) | MAPK14 | Q16539 | √ |
| MOL002914 | Eriodyctiol (flavanone) | MMP2 | P08253 | √ |
| MOL002914 | Eriodyctiol (flavanone) | PGD | P52209 | √ |
| MOL002914 | Eriodyctiol (flavanone) | ST3GAL3 | Q11203 | √ |
| MOL002914 | Eriodyctiol (flavanone) | FUT7 | Q11130 | √ |
| MOL002914 | Eriodyctiol (flavanone) | FUT4 | P22083 | √ |
| MOL002914 | Eriodyctiol (flavanone) | STAT1 | P42224 | √ |
| MOL002914 | Eriodyctiol (flavanone) | SQLE | Q14534 | √ |
| MOL002914 | Eriodyctiol (flavanone) | RXRA | P19793 | √ |
| MOL002914 | Eriodyctiol (flavanone) | CHRNA7 | P36544 | √ |
| MOL002914 | Eriodyctiol (flavanone) | GSK3B | P49841 | √ |
| MOL002914 | Eriodyctiol (flavanone) | KIT | P10721 | √ |
| MOL002914 | Eriodyctiol (flavanone) | KDR | P35968 | √ |
| MOL002914 | Eriodyctiol (flavanone) | FGFR1 | P11362 | √ |
| MOL002914 | Eriodyctiol (flavanone) | MET | P08581 | √ |
| MOL002914 | Eriodyctiol (flavanone) | GRM2 | Q14416 | √ |
| MOL002914 | Eriodyctiol (flavanone) | MMP9 | P14780 | √ |
| MOL002914 | Eriodyctiol (flavanone) | FFAR1 | O14842 | √ |
| MOL002914 | Eriodyctiol (flavanone) | HIF1A | Q16665 | √ |
| MOL002914 | Eriodyctiol (flavanone) | CTSB | P07858 | √ |
| MOL002914 | Eriodyctiol (flavanone) | SERPINE1 | P05121 | √ |
| MOL002914 | Eriodyctiol (flavanone) | DYRK1A | Q13627 | √ |
| MOL002914 | Eriodyctiol (flavanone) | CTSL | P07711 | √ |
| MOL002914 | Eriodyctiol (flavanone) | YWHAG | P61981 | √ |
| MOL002914 | Eriodyctiol (flavanone) | ODC1 | P11926 | √ |
| MOL002914 | Eriodyctiol (flavanone) | CA7 | P43166 | × |
| MOL002914 | Eriodyctiol (flavanone) | CA12 | O43570 | × |
| MOL002914 | Eriodyctiol (flavanone) | CA4 | P22748 | × |
| MOL002914 | Eriodyctiol (flavanone) | ABCC1 | P33527 | × |
| MOL002914 | Eriodyctiol (flavanone) | CBR1 | P16152 | × |
| MOL002914 | Eriodyctiol (flavanone) | ADORA3 | P0DMS8 | × |
| MOL002914 | Eriodyctiol (flavanone) | ADORA1 | P30542 | × |
| MOL002914 | Eriodyctiol (flavanone) | ABCG2 | Q9UNQ0 | × |
| MOL002914 | Eriodyctiol (flavanone) | AKR1C3 | P42330 | × |
| MOL002914 | Eriodyctiol (flavanone) | ACHE | P22303 | × |
| MOL002914 | Eriodyctiol (flavanone) | ABCB1 | P08183 | × |
| MOL002914 | Eriodyctiol (flavanone) | BACE1 | P56817 | × |
| MOL002914 | Eriodyctiol (flavanone) | BCHE | P06276 | × |
| MOL002914 | Eriodyctiol (flavanone) | CA2 | P00918 | × |
| MOL002914 | Eriodyctiol (flavanone) | CA1 | P00915 | × |
| MOL002914 | Eriodyctiol (flavanone) | ALOX12 | P18054 | × |
| MOL002914 | Eriodyctiol (flavanone) | CA3 | P07451 | × |
| MOL002914 | Eriodyctiol (flavanone) | CA6 | P23280 | × |
| MOL002914 | Eriodyctiol (flavanone) | CA13 | Q8N1Q1 | × |
| MOL002914 | Eriodyctiol (flavanone) | CA5B | Q9Y2D0 | × |
| MOL002914 | Eriodyctiol (flavanone) | CA5A | P35218 | × |
| MOL002914 | Eriodyctiol (flavanone) | CA9 | Q16790 | × |
| MOL002914 | Eriodyctiol (flavanone) | APP | P05067 | × |
| MOL002914 | Eriodyctiol (flavanone) | AKT1 | P31749 | × |
| MOL003006 | (-)-(3R,8S,9R,9aS,10aS)-9-ethenyl-8-(beta-D-glucopyranosyloxy)-2,3,9,9a,10,10a-hexahydro-5-oxo-5H,8H-pyrano[4,3-d]oxazolo[3,2-a]pyridine-3-carboxylic acid_qt | ECE1 | P42892 | √ |
| MOL003006 | (-)-(3R,8S,9R,9aS,10aS)-9-ethenyl-8-(beta-D-glucopyranosyloxy)-2,3,9,9a,10,10a-hexahydro-5-oxo-5H,8H-pyrano[4,3-d]oxazolo[3,2-a]pyridine-3-carboxylic acid_qt | MME | P08473 | √ |
| MOL003006 | (-)-(3R,8S,9R,9aS,10aS)-9-ethenyl-8-(beta-D-glucopyranosyloxy)-2,3,9,9a,10,10a-hexahydro-5-oxo-5H,8H-pyrano[4,3-d]oxazolo[3,2-a]pyridine-3-carboxylic acid_qt | DNMT3B | Q9UBC3 | √ |
| MOL003006 | (-)-(3R,8S,9R,9aS,10aS)-9-ethenyl-8-(beta-D-glucopyranosyloxy)-2,3,9,9a,10,10a-hexahydro-5-oxo-5H,8H-pyrano[4,3-d]oxazolo[3,2-a]pyridine-3-carboxylic acid_qt | REN | P00797 | √ |
| MOL003006 | (-)-(3R,8S,9R,9aS,10aS)-9-ethenyl-8-(beta-D-glucopyranosyloxy)-2,3,9,9a,10,10a-hexahydro-5-oxo-5H,8H-pyrano[4,3-d]oxazolo[3,2-a]pyridine-3-carboxylic acid_qt | SLC13A5 | Q86YT5 | √ |
| MOL003006 | (-)-(3R,8S,9R,9aS,10aS)-9-ethenyl-8-(beta-D-glucopyranosyloxy)-2,3,9,9a,10,10a-hexahydro-5-oxo-5H,8H-pyrano[4,3-d]oxazolo[3,2-a]pyridine-3-carboxylic acid_qt | SELL | P14151 | √ |
| MOL003006 | (-)-(3R,8S,9R,9aS,10aS)-9-ethenyl-8-(beta-D-glucopyranosyloxy)-2,3,9,9a,10,10a-hexahydro-5-oxo-5H,8H-pyrano[4,3-d]oxazolo[3,2-a]pyridine-3-carboxylic acid_qt | SELP | P16109 | √ |
| MOL003006 | (-)-(3R,8S,9R,9aS,10aS)-9-ethenyl-8-(beta-D-glucopyranosyloxy)-2,3,9,9a,10,10a-hexahydro-5-oxo-5H,8H-pyrano[4,3-d]oxazolo[3,2-a]pyridine-3-carboxylic acid_qt | ENGASE | Q8NFI3 | √ |
| MOL003006 | (-)-(3R,8S,9R,9aS,10aS)-9-ethenyl-8-(beta-D-glucopyranosyloxy)-2,3,9,9a,10,10a-hexahydro-5-oxo-5H,8H-pyrano[4,3-d]oxazolo[3,2-a]pyridine-3-carboxylic acid_qt | FTO | Q9C0B1 | √ |
| MOL003006 | (-)-(3R,8S,9R,9aS,10aS)-9-ethenyl-8-(beta-D-glucopyranosyloxy)-2,3,9,9a,10,10a-hexahydro-5-oxo-5H,8H-pyrano[4,3-d]oxazolo[3,2-a]pyridine-3-carboxylic acid_qt | AMPD3 | Q01432 | × |
| MOL003006 | (-)-(3R,8S,9R,9aS,10aS)-9-ethenyl-8-(beta-D-glucopyranosyloxy)-2,3,9,9a,10,10a-hexahydro-5-oxo-5H,8H-pyrano[4,3-d]oxazolo[3,2-a]pyridine-3-carboxylic acid_qt | ACE | P12821 | × |
| MOL003006 | (-)-(3R,8S,9R,9aS,10aS)-9-ethenyl-8-(beta-D-glucopyranosyloxy)-2,3,9,9a,10,10a-hexahydro-5-oxo-5H,8H-pyrano[4,3-d]oxazolo[3,2-a]pyridine-3-carboxylic acid_qt | ACLY | P53396 | × |
| MOL003006 | (-)-(3R,8S,9R,9aS,10aS)-9-ethenyl-8-(beta-D-glucopyranosyloxy)-2,3,9,9a,10,10a-hexahydro-5-oxo-5H,8H-pyrano[4,3-d]oxazolo[3,2-a]pyridine-3-carboxylic acid_qt | CA2 | P00918 | × |
| MOL003006 | (-)-(3R,8S,9R,9aS,10aS)-9-ethenyl-8-(beta-D-glucopyranosyloxy)-2,3,9,9a,10,10a-hexahydro-5-oxo-5H,8H-pyrano[4,3-d]oxazolo[3,2-a]pyridine-3-carboxylic acid_qt | CA1 | P00915 | × |
| MOL003006 | (-)-(3R,8S,9R,9aS,10aS)-9-ethenyl-8-(beta-D-glucopyranosyloxy)-2,3,9,9a,10,10a-hexahydro-5-oxo-5H,8H-pyrano[4,3-d]oxazolo[3,2-a]pyridine-3-carboxylic acid_qt | AKR1B1 | P15121 | × |
| MOL003006 | (-)-(3R,8S,9R,9aS,10aS)-9-ethenyl-8-(beta-D-glucopyranosyloxy)-2,3,9,9a,10,10a-hexahydro-5-oxo-5H,8H-pyrano[4,3-d]oxazolo[3,2-a]pyridine-3-carboxylic acid_qt | CASP3 | P42574 | × |
| MOL003006 | (-)-(3R,8S,9R,9aS,10aS)-9-ethenyl-8-(beta-D-glucopyranosyloxy)-2,3,9,9a,10,10a-hexahydro-5-oxo-5H,8H-pyrano[4,3-d]oxazolo[3,2-a]pyridine-3-carboxylic acid_qt | CASP6 | P55212 | × |
| MOL003006 | (-)-(3R,8S,9R,9aS,10aS)-9-ethenyl-8-(beta-D-glucopyranosyloxy)-2,3,9,9a,10,10a-hexahydro-5-oxo-5H,8H-pyrano[4,3-d]oxazolo[3,2-a]pyridine-3-carboxylic acid_qt | CASP7 | P55210 | × |
| MOL003006 | (-)-(3R,8S,9R,9aS,10aS)-9-ethenyl-8-(beta-D-glucopyranosyloxy)-2,3,9,9a,10,10a-hexahydro-5-oxo-5H,8H-pyrano[4,3-d]oxazolo[3,2-a]pyridine-3-carboxylic acid_qt | CASP8 | Q14790 | × |
| MOL003006 | (-)-(3R,8S,9R,9aS,10aS)-9-ethenyl-8-(beta-D-glucopyranosyloxy)-2,3,9,9a,10,10a-hexahydro-5-oxo-5H,8H-pyrano[4,3-d]oxazolo[3,2-a]pyridine-3-carboxylic acid_qt | CASP1 | P29466 | × |
| MOL003006 | (-)-(3R,8S,9R,9aS,10aS)-9-ethenyl-8-(beta-D-glucopyranosyloxy)-2,3,9,9a,10,10a-hexahydro-5-oxo-5H,8H-pyrano[4,3-d]oxazolo[3,2-a]pyridine-3-carboxylic acid_qt | CASP2 | P42575 | × |
| MOL003014 | secologanic dibutylacetal_qt | PYGL | P06737 | √ |
| MOL003014 | secologanic dibutylacetal_qt | CFD | P00746 | √ |
| MOL003014 | secologanic dibutylacetal_qt | MAPK14 | Q16539 | √ |
| MOL003014 | secologanic dibutylacetal_qt | STAT3 | P40763 | √ |
| MOL003014 | secologanic dibutylacetal_qt | DHODH | Q02127 | √ |
| MOL003014 | secologanic dibutylacetal_qt | EGFR | P00533 | √ |
| MOL003014 | secologanic dibutylacetal_qt | PRKCA | P17252 | √ |
| MOL003014 | secologanic dibutylacetal_qt | RASGRP3 | Q8IV61 | √ |
| MOL003014 | secologanic dibutylacetal_qt | GRM5 | P41594 | √ |
| MOL003014 | secologanic dibutylacetal_qt | PDE10A | Q9Y233 | √ |
| MOL003014 | secologanic dibutylacetal_qt | PTGS2 | P35354 | √ |
| MOL003014 | secologanic dibutylacetal_qt | MET | P08581 | √ |
| MOL003014 | secologanic dibutylacetal_qt | KCNA3 | P22001 | √ |
| MOL003014 | secologanic dibutylacetal_qt | CNR1 | P21554 | √ |
| MOL003014 | secologanic dibutylacetal_qt | FAAH | O00519 | √ |
| MOL003014 | secologanic dibutylacetal_qt | CDK1 | P06493 | √ |
| MOL003014 | secologanic dibutylacetal_qt | CTSS | P25774 | √ |
| MOL003014 | secologanic dibutylacetal_qt | MMP13 | P45452 | √ |
| MOL003014 | secologanic dibutylacetal_qt | P2RX3 | P56373 | √ |
| MOL003014 | secologanic dibutylacetal_qt | MMP1 | P03956 | √ |
| MOL003014 | secologanic dibutylacetal_qt | IRAK4 | Q9NWZ3 | √ |
| MOL003014 | secologanic dibutylacetal_qt | GABRA5 | P31644 | √ |
| MOL003014 | secologanic dibutylacetal_qt | ERBB2 | P04626 | √ |
| MOL003014 | secologanic dibutylacetal_qt | EDNRA | P25101 | √ |
| MOL003014 | secologanic dibutylacetal_qt | CTSK | P43235 | √ |
| MOL003014 | secologanic dibutylacetal_qt | PDE4B | Q07343 | √ |
| MOL003014 | secologanic dibutylacetal_qt | CHRM1 | P11229 | √ |
| MOL003014 | secologanic dibutylacetal_qt | SLC33A1 | O00400 | √ |
| MOL003014 | secologanic dibutylacetal_qt | HTR1A | P08908 | √ |
| MOL003014 | secologanic dibutylacetal_qt | DRD2 | P14416 | √ |
| MOL003014 | secologanic dibutylacetal_qt | HRH1 | P35367 | √ |
| MOL003014 | secologanic dibutylacetal_qt | MTOR | P42345 | √ |
| MOL003014 | secologanic dibutylacetal_qt | HTR7 | P34969 | √ |
| MOL003014 | secologanic dibutylacetal_qt | LTB4R | Q15722 | √ |
| MOL003014 | secologanic dibutylacetal_qt | PIK3CA | P42336 | √ |
| MOL003014 | secologanic dibutylacetal_qt | SMO | Q99835 | √ |
| MOL003014 | secologanic dibutylacetal_qt | KCNH2 | Q12809 | √ |
| MOL003014 | secologanic dibutylacetal_qt | FFAR1 | O14842 | √ |
| MOL003014 | secologanic dibutylacetal_qt | MMP9 | P14780 | √ |
| MOL003014 | secologanic dibutylacetal_qt | MMP12 | P39900 | √ |
| MOL003014 | secologanic dibutylacetal_qt | HCRTR2 | O43614 | √ |
| MOL003014 | secologanic dibutylacetal_qt | HCRTR1 | O43613 | √ |
| MOL003014 | secologanic dibutylacetal_qt | RET | P07949 | √ |
| MOL003014 | secologanic dibutylacetal_qt | KDR | P35968 | √ |
| MOL003014 | secologanic dibutylacetal_qt | LIPG | Q9Y5X9 | √ |
| MOL003014 | secologanic dibutylacetal_qt | JAK3 | P52333 | √ |
| MOL003014 | secologanic dibutylacetal_qt | JAK1 | P23458 | √ |
| MOL003014 | secologanic dibutylacetal_qt | MMP3 | P08254 | √ |
| MOL003014 | secologanic dibutylacetal_qt | JAK2 | O60674 | √ |
| MOL003014 | secologanic dibutylacetal_qt | MMP2 | P08253 | √ |
| MOL003014 | secologanic dibutylacetal_qt | TYK2 | P29597 | √ |
| MOL003014 | secologanic dibutylacetal_qt | CTSL | P07711 | √ |
| MOL003014 | secologanic dibutylacetal_qt | MMP8 | P22894 | √ |
| MOL003014 | secologanic dibutylacetal_qt | GSK3B | P49841 | √ |
| MOL003014 | secologanic dibutylacetal_qt | FBP1 | P09467 | √ |
| MOL003014 | secologanic dibutylacetal_qt | CSF1R | P07333 | √ |
| MOL003014 | secologanic dibutylacetal_qt | CDC7 | O00311 | √ |
| MOL003014 | secologanic dibutylacetal_qt | WNT3A | P56704 | √ |
| MOL003014 | secologanic dibutylacetal_qt | PIN1 | Q13526 | √ |
| MOL003014 | secologanic dibutylacetal_qt | DYRK1A | Q13627 | √ |
| MOL003014 | secologanic dibutylacetal_qt | DRD3 | P35462 | √ |
| MOL003014 | secologanic dibutylacetal_qt | PTGER3 | P43115 | √ |
| MOL003014 | secologanic dibutylacetal_qt | MAPK1 | P28482 | √ |
| MOL003014 | secologanic dibutylacetal_qt | TNKS2 | Q9H2K2 | √ |
| MOL003014 | secologanic dibutylacetal_qt | PDE5A | O76074 | √ |
| MOL003014 | secologanic dibutylacetal_qt | CXCR2 | P25025 | √ |
| MOL003014 | secologanic dibutylacetal_qt | F10 | P00742 | √ |
| MOL003014 | secologanic dibutylacetal_qt | CDK2 | P24941 | √ |
| MOL003014 | secologanic dibutylacetal_qt | CDK9 | P50750 | √ |
| MOL003014 | secologanic dibutylacetal_qt | CHRNA7 | P36544 | √ |
| MOL003014 | secologanic dibutylacetal_qt | NPY5R | Q15761 | √ |
| MOL003014 | secologanic dibutylacetal_qt | IDO1 | P14902 | √ |
| MOL003014 | secologanic dibutylacetal_qt | SYK | P43405 | √ |
| MOL003014 | secologanic dibutylacetal_qt | PSEN2 | P49810 | √ |
| MOL003014 | secologanic dibutylacetal_qt | PSENEN | Q9NZ42 | √ |
| MOL003014 | secologanic dibutylacetal_qt | NCSTN | Q92542 | √ |
| MOL003014 | secologanic dibutylacetal_qt | PSEN1 | P49768 | √ |
| MOL003014 | secologanic dibutylacetal_qt | FNTA | P49354 | √ |
| MOL003014 | secologanic dibutylacetal_qt | FNTB | P49356 | √ |
| MOL003014 | secologanic dibutylacetal_qt | PIK3CA | P42336 | √ |
| MOL003014 | secologanic dibutylacetal_qt | PIK3R1 | P27986 | √ |
| MOL003014 | secologanic dibutylacetal_qt | CDK2 | P24941 | √ |
| MOL003014 | secologanic dibutylacetal_qt | CDK4 | P11802 | √ |
| MOL003014 | secologanic dibutylacetal_qt | CDK1 | P06493 | √ |
| MOL003014 | secologanic dibutylacetal_qt | CDK2 | P24941 | √ |
| MOL003014 | secologanic dibutylacetal_qt | CDK5R1 | Q15078 | √ |
| MOL003014 | secologanic dibutylacetal_qt | CDK5 | Q00535 | √ |
| MOL003014 | secologanic dibutylacetal_qt | CDK9 | P50750 | √ |
| MOL003014 | secologanic dibutylacetal_qt | CASP3 | P42574 | × |
| MOL003014 | secologanic dibutylacetal_qt | CASP8 | Q14790 | × |
| MOL003014 | secologanic dibutylacetal_qt | CASP1 | P29466 | × |
| MOL003014 | secologanic dibutylacetal_qt | BDKRB1 | P46663 | × |
| MOL003014 | secologanic dibutylacetal_qt | CASP7 | P55210 | × |
| MOL003014 | secologanic dibutylacetal_qt | ABCC9 | O60706 | × |
| MOL003014 | secologanic dibutylacetal_qt | CASP6 | P55212 | × |
| MOL003014 | secologanic dibutylacetal_qt | ADAM17 | P78536 | × |
| MOL003014 | secologanic dibutylacetal_qt | CAPN1 | P07384 | × |
| MOL003014 | secologanic dibutylacetal_qt | ADORA1 | P30542 | × |
| MOL003014 | secologanic dibutylacetal_qt | ABCB1 | P08183 | × |
| MOL003014 | secologanic dibutylacetal_qt | AR | P10275 | × |
| MOL003014 | secologanic dibutylacetal_qt | BACE1 | P56817 | × |
| MOL003014 | secologanic dibutylacetal_qt | CCR3 | P51677 | × |
| MOL003014 | secologanic dibutylacetal_qt | ADCY1 | Q08828 | × |
| MOL003014 | secologanic dibutylacetal_qt | ADAM10 | O14672 | × |
| MOL003014 | secologanic dibutylacetal_qt | ADORA2A | P29274 | × |
| MOL003014 | secologanic dibutylacetal_qt | ALOX5 | P09917 | × |
| MOL003014 | secologanic dibutylacetal_qt | APH1A | Q96BI3 | × |
| MOL003014 | secologanic dibutylacetal_qt | APH1B | Q8WW43 | × |
| MOL003014 | secologanic dibutylacetal_qt | CCNA1 | P78396 | × |
| MOL003014 | secologanic dibutylacetal_qt | CCNA2 | P20248 | × |
| MOL003014 | secologanic dibutylacetal_qt | CCND1 | P24385 | × |
| MOL003014 | secologanic dibutylacetal_qt | CCNB1 | P14635 | × |
| MOL003014 | secologanic dibutylacetal_qt | CCNE1 | P24864 | × |
| MOL003014 | secologanic dibutylacetal_qt | CCK7 | P50613 | × |
| MOL003014 | secologanic dibutylacetal_qt | CCNH | P51946 | × |
| MOL003014 | secologanic dibutylacetal_qt | CCNT1 | O60563 | × |
| MOL003036 | ZINC03978781 | NPC1L1 | Q9UHC9 | √ |
| MOL003036 | ZINC03978781 | NR1H3 | Q13133 | √ |
| MOL003036 | ZINC03978781 | RORC | P51449 | √ |
| MOL003036 | ZINC03978781 | HMGCR | P04035 | √ |
| MOL003036 | ZINC03978781 | CYP51A1 | Q16850 | √ |
| MOL003036 | ZINC03978781 | CYP17A1 | P05093 | √ |
| MOL003036 | ZINC03978781 | SREBF2 | Q12772 | √ |
| MOL003036 | ZINC03978781 | SHBG | P04278 | √ |
| MOL003036 | ZINC03978781 | RORA | P35398 | √ |
| MOL003036 | ZINC03978781 | ESR1 | P03372 | √ |
| MOL003036 | ZINC03978781 | ESR2 | Q92731 | √ |
| MOL003036 | ZINC03978781 | CYP19A1 | P11511 | √ |
| MOL003036 | ZINC03978781 | SLC6A2 | P23975 | √ |
| MOL003036 | ZINC03978781 | PTPN1 | P18031 | √ |
| MOL003036 | ZINC03978781 | CYP2C19 | P33261 | √ |
| MOL003036 | ZINC03978781 | SERPINA6 | P08185 | √ |
| MOL003036 | ZINC03978781 | CHRM2 | P08172 | √ |
| MOL003036 | ZINC03978781 | NR1H2 | P55055 | √ |
| MOL003036 | ZINC03978781 | SLC6A4 | P31645 | √ |
| MOL003036 | ZINC03978781 | VDR | P11473 | √ |
| MOL003036 | ZINC03978781 | NR1I3 | Q14994 | √ |
| MOL003036 | ZINC03978781 | DHCR7 | Q9UBM7 | √ |
| MOL003036 | ZINC03978781 | G6PD | P11413 | √ |
| MOL003036 | ZINC03978781 | GLRA1 | P23415 | √ |
| MOL003036 | ZINC03978781 | HSD11B1 | P28845 | √ |
| MOL003036 | ZINC03978781 | CES2 | O00748 | √ |
| MOL003036 | ZINC03978781 | PTGER1 | P34995 | √ |
| MOL003036 | ZINC03978781 | PTGER2 | P43116 | √ |
| MOL003036 | ZINC03978781 | PTGES | O14684 | √ |
| MOL003036 | ZINC03978781 | NOS2 | P35228 | √ |
| MOL003036 | ZINC03978781 | PPARD | Q03181 | √ |
| MOL003036 | ZINC03978781 | SQLE | Q14534 | √ |
| MOL003036 | ZINC03978781 | PTPN6 | P29350 | √ |
| MOL003036 | ZINC03978781 | PTPN2 | P17706 | √ |
| MOL003036 | ZINC03978781 | HSD11B2 | P80365 | √ |
| MOL003036 | ZINC03978781 | MDM2 | Q00987 | √ |
| MOL003036 | ZINC03978781 | UGT2B7 | P16662 | √ |
| MOL003036 | ZINC03978781 | POLB | P06746 | √ |
| MOL003036 | ZINC03978781 | AR | P10275 | × |
| MOL003036 | ZINC03978781 | BCHE | P06276 | × |
| MOL003036 | ZINC03978781 | ACHE | P22303 | × |
| MOL003044 | Chryseriol | CYP1B1 | Q16678 | √ |
| MOL003044 | Chryseriol | XDH | P47989 | √ |
| MOL003044 | Chryseriol | PTPRS | Q13332 | √ |
| MOL003044 | Chryseriol | PLG | P00747 | √ |
| MOL003044 | Chryseriol | PARP1 | P09874 | √ |
| MOL003044 | Chryseriol | TNKS2 | Q9H2K2 | √ |
| MOL003044 | Chryseriol | TNKS | O95271 | √ |
| MOL003044 | Chryseriol | NOX4 | Q9NPH5 | √ |
| MOL003044 | Chryseriol | FLT3 | P36888 | √ |
| MOL003044 | Chryseriol | MAOA | P21397 | √ |
| MOL003044 | Chryseriol | GLO1 | Q04760 | √ |
| MOL003044 | Chryseriol | SYK | P43405 | √ |
| MOL003044 | Chryseriol | GSK3B | P49841 | √ |
| MOL003044 | Chryseriol | TTR | P02766 | √ |
| MOL003044 | Chryseriol | MMP9 | P14780 | √ |
| MOL003044 | Chryseriol | MMP2 | P08253 | √ |
| MOL003044 | Chryseriol | MMP12 | P39900 | √ |
| MOL003044 | Chryseriol | CD38 | P28907 | √ |
| MOL003044 | Chryseriol | TOP1 | P11387 | √ |
| MOL003044 | Chryseriol | ESR2 | Q92731 | √ |
| MOL003044 | Chryseriol | CDK6 | Q00534 | √ |
| MOL003044 | Chryseriol | PLA2G2A | P14555 | √ |
| MOL003044 | Chryseriol | PIM1 | P11309 | √ |
| MOL003044 | Chryseriol | TERT | O14746 | √ |
| MOL003044 | Chryseriol | HSD17B1 | P14061 | √ |
| MOL003044 | Chryseriol | ESR1 | P03372 | √ |
| MOL003044 | Chryseriol | CYP19A1 | P11511 | √ |
| MOL003044 | Chryseriol | CSNK2A1 | P68400 | √ |
| MOL003044 | Chryseriol | IGF1R | P08069 | √ |
| MOL003044 | Chryseriol | EGFR | P00533 | √ |
| MOL003044 | Chryseriol | OPRD1 | P41143 | √ |
| MOL003044 | Chryseriol | CDK2 | P24941 | √ |
| MOL003044 | Chryseriol | PTGS2 | P35354 | √ |
| MOL003044 | Chryseriol | CFTR | P13569 | √ |
| MOL003044 | Chryseriol | MCL1 | Q07820 | √ |
| MOL003044 | Chryseriol | HSD17B2 | P37059 | √ |
| MOL003044 | Chryseriol | F2 | P00734 | √ |
| MOL003044 | Chryseriol | CDK1 | P06493 | √ |
| MOL003044 | Chryseriol | DRD4 | P21917 | √ |
| MOL003044 | Chryseriol | MPO | P05164 | √ |
| MOL003044 | Chryseriol | PIK3R1 | P27986 | √ |
| MOL003044 | Chryseriol | DAPK1 | P53355 | √ |
| MOL003044 | Chryseriol | PYGL | P06737 | √ |
| MOL003044 | Chryseriol | SRC | P12931 | √ |
| MOL003044 | Chryseriol | PTK2 | Q05397 | √ |
| MOL003044 | Chryseriol | KDR | P35968 | √ |
| MOL003044 | Chryseriol | MMP13 | P45452 | √ |
| MOL003044 | Chryseriol | MMP3 | P08254 | √ |
| MOL003044 | Chryseriol | PLK1 | P53350 | √ |
| MOL003044 | Chryseriol | PKN1 | Q16512 | √ |
| MOL003044 | Chryseriol | MET | P08581 | √ |
| MOL003044 | Chryseriol | NEK2 | P51955 | √ |
| MOL003044 | Chryseriol | CXCR1 | P25024 | √ |
| MOL003044 | Chryseriol | NEK6 | Q9HC98 | √ |
| MOL003044 | Chryseriol | PLA2G1B | P04054 | √ |
| MOL003044 | Chryseriol | NUAK1 | O60285 | √ |
| MOL003044 | Chryseriol | GPR35 | Q9HC97 | √ |
| MOL003044 | Chryseriol | GRK6 | P43250 | √ |
| MOL003044 | Chryseriol | TYR | P14679 | √ |
| MOL003044 | Chryseriol | ST6GAL1 | P15907 | √ |
| MOL003044 | Chryseriol | CDK5R1 | Q15078 | √ |
| MOL003044 | Chryseriol | CDK5 | Q00535 | √ |
| MOL003044 | Chryseriol | CDK1 | P06493 | √ |
| MOL003044 | Chryseriol | ABCC1 | P33527 | × |
| MOL003044 | Chryseriol | AKR1B1 | P15121 | × |
| MOL003044 | Chryseriol | CA2 | P00918 | × |
| MOL003044 | Chryseriol | CA7 | P43166 | × |
| MOL003044 | Chryseriol | CA12 | O43570 | × |
| MOL003044 | Chryseriol | CA4 | P22748 | × |
| MOL003044 | Chryseriol | ARG1 | P05089 | × |
| MOL003044 | Chryseriol | APP | P05067 | × |
| MOL003044 | Chryseriol | ALOX5 | P09917 | × |
| MOL003044 | Chryseriol | ABCB1 | P08183 | × |
| MOL003044 | Chryseriol | ABCG2 | Q9UNQ0 | × |
| MOL003044 | Chryseriol | ADORA1 | P30542 | × |
| MOL003044 | Chryseriol | AKR1B10 | O60218 | × |
| MOL003044 | Chryseriol | ACHE | P22303 | × |
| MOL003044 | Chryseriol | ADORA2A | P29274 | × |
| MOL003044 | Chryseriol | ALOX15 | P16050 | × |
| MOL003044 | Chryseriol | ALOX12 | P18054 | × |
| MOL003044 | Chryseriol | CBR1 | P16152 | × |
| MOL003044 | Chryseriol | CA1 | P00915 | × |
| MOL003044 | Chryseriol | CA9 | Q16790 | × |
| MOL003044 | Chryseriol | AVPR2 | P30518 | × |
| MOL003044 | Chryseriol | AURKB | Q96GD4 | × |
| MOL003044 | Chryseriol | CA3 | P07451 | × |
| MOL003044 | Chryseriol | CA6 | P23280 | × |
| MOL003044 | Chryseriol | CA14 | Q9ULX7 | × |
| MOL003044 | Chryseriol | CAMK2B | Q13554 | × |
| MOL003044 | Chryseriol | ALK | Q9UM73 | × |
| MOL003044 | Chryseriol | AKT1 | P31749 | × |
| MOL003044 | Chryseriol | CA5A | P35218 | × |
| MOL003044 | Chryseriol | BACE1 | P56817 | × |
| MOL003044 | Chryseriol | AXL | P30530 | × |
| MOL003044 | Chryseriol | AKR1C2 | P52895 | × |
| MOL003044 | Chryseriol | AKR1C1 | Q04828 | × |
| MOL003044 | Chryseriol | AKR1C3 | P42330 | × |
| MOL003044 | Chryseriol | AKR1C4 | P17516 | × |
| MOL003044 | Chryseriol | CA13 | Q8N1Q1 | × |
| MOL003044 | Chryseriol | AKR1A1 | P14550 | × |
| MOL003044 | Chryseriol | AMY1A | P04745 | × |
| MOL003044 | Chryseriol | CCNB3 | Q8WWL7 | × |
| MOL003044 | Chryseriol | CCNB1 | P14635 | × |
| MOL003044 | Chryseriol | CCNB2 | O95067 | × |
| MOL003059 | kryptoxanthin | PTPN1 | P18031 | √ |
| MOL003059 | kryptoxanthin | NR1H3 | Q13133 | √ |
| MOL003059 | kryptoxanthin | VDR | P11473 | √ |
| MOL003059 | kryptoxanthin | CDC25A | P30304 | √ |
| MOL003059 | kryptoxanthin | GLRA1 | P23415 | √ |
| MOL003059 | kryptoxanthin | SLC6A2 | P23975 | √ |
| MOL003059 | kryptoxanthin | GC | P02774 | √ |
| MOL003059 | kryptoxanthin | CYP19A1 | P11511 | √ |
| MOL003059 | kryptoxanthin | ESR1 | P03372 | √ |
| MOL003059 | kryptoxanthin | ESR2 | Q92731 | √ |
| MOL003059 | kryptoxanthin | RBP4 | P02753 | √ |
| MOL003059 | kryptoxanthin | RORC | P51449 | √ |
| MOL003059 | kryptoxanthin | SREBF2 | Q12772 | √ |
| MOL003059 | kryptoxanthin | NPC1L1 | Q9UHC9 | √ |
| MOL003059 | kryptoxanthin | SHBG | P04278 | √ |
| MOL003059 | kryptoxanthin | CYP17A1 | P05093 | √ |
| MOL003059 | kryptoxanthin | HMGCR | P04035 | √ |
| MOL003059 | kryptoxanthin | CYP51A1 | Q16850 | √ |
| MOL003059 | kryptoxanthin | AR | P10275 | × |
| MOL003062 | 4,5'-Retro-.beta.,.beta.-Carotene-3,3'-dione, 4',5'-didehydro- | PGR | P06401 | √ |
| MOL003062 | 4,5'-Retro-.beta.,.beta.-Carotene-3,3'-dione, 4',5'-didehydro- | CYP19A1 | P11511 | √ |
| MOL003062 | 4,5'-Retro-.beta.,.beta.-Carotene-3,3'-dione, 4',5'-didehydro- | NR3C1 | P04150 | √ |
| MOL003062 | 4,5'-Retro-.beta.,.beta.-Carotene-3,3'-dione, 4',5'-didehydro- | NR3C2 | P08235 | √ |
| MOL003062 | 4,5'-Retro-.beta.,.beta.-Carotene-3,3'-dione, 4',5'-didehydro- | SHBG | P04278 | √ |
| MOL003062 | 4,5'-Retro-.beta.,.beta.-Carotene-3,3'-dione, 4',5'-didehydro- | HAO1 | Q9UJM8 | √ |
| MOL003062 | 4,5'-Retro-.beta.,.beta.-Carotene-3,3'-dione, 4',5'-didehydro- | CES2 | O00748 | √ |
| MOL003062 | 4,5'-Retro-.beta.,.beta.-Carotene-3,3'-dione, 4',5'-didehydro- | SERPINA6 | P08185 | √ |
| MOL003062 | 4,5'-Retro-.beta.,.beta.-Carotene-3,3'-dione, 4',5'-didehydro- | RARG | P13631 | √ |
| MOL003062 | 4,5'-Retro-.beta.,.beta.-Carotene-3,3'-dione, 4',5'-didehydro- | RXRG | P48443 | √ |
| MOL003062 | 4,5'-Retro-.beta.,.beta.-Carotene-3,3'-dione, 4',5'-didehydro- | RARB | P10826 | √ |
| MOL003062 | 4,5'-Retro-.beta.,.beta.-Carotene-3,3'-dione, 4',5'-didehydro- | RARA | P10276 | √ |
| MOL003062 | 4,5'-Retro-.beta.,.beta.-Carotene-3,3'-dione, 4',5'-didehydro- | RXRA | P19793 | √ |
| MOL003062 | 4,5'-Retro-.beta.,.beta.-Carotene-3,3'-dione, 4',5'-didehydro- | AR | P10275 | × |
| MOL003095 | 5-hydroxy-7-methoxy-2-(3,4,5-trimethoxyphenyl)chromone | OPRD1 | P41143 | √ |
| MOL003095 | 5-hydroxy-7-methoxy-2-(3,4,5-trimethoxyphenyl)chromone | CYP1B1 | Q16678 | √ |
| MOL003095 | 5-hydroxy-7-methoxy-2-(3,4,5-trimethoxyphenyl)chromone | PLG | P00747 | √ |
| MOL003095 | 5-hydroxy-7-methoxy-2-(3,4,5-trimethoxyphenyl)chromone | PLA2G2A | P14555 | √ |
| MOL003095 | 5-hydroxy-7-methoxy-2-(3,4,5-trimethoxyphenyl)chromone | MMP9 | P14780 | √ |
| MOL003095 | 5-hydroxy-7-methoxy-2-(3,4,5-trimethoxyphenyl)chromone | MMP2 | P08253 | √ |
| MOL003095 | 5-hydroxy-7-methoxy-2-(3,4,5-trimethoxyphenyl)chromone | MMP12 | P39900 | √ |
| MOL003095 | 5-hydroxy-7-methoxy-2-(3,4,5-trimethoxyphenyl)chromone | NOX4 | Q9NPH5 | √ |
| MOL003095 | 5-hydroxy-7-methoxy-2-(3,4,5-trimethoxyphenyl)chromone | XDH | P47989 | √ |
| MOL003095 | 5-hydroxy-7-methoxy-2-(3,4,5-trimethoxyphenyl)chromone | SYK | P43405 | √ |
| MOL003095 | 5-hydroxy-7-methoxy-2-(3,4,5-trimethoxyphenyl)chromone | PARP1 | P09874 | √ |
| MOL003095 | 5-hydroxy-7-methoxy-2-(3,4,5-trimethoxyphenyl)chromone | TTR | P02766 | √ |
| MOL003095 | 5-hydroxy-7-methoxy-2-(3,4,5-trimethoxyphenyl)chromone | CD38 | P28907 | √ |
| MOL003095 | 5-hydroxy-7-methoxy-2-(3,4,5-trimethoxyphenyl)chromone | TOP1 | P11387 | √ |
| MOL003095 | 5-hydroxy-7-methoxy-2-(3,4,5-trimethoxyphenyl)chromone | PTPRS | Q13332 | √ |
| MOL003095 | 5-hydroxy-7-methoxy-2-(3,4,5-trimethoxyphenyl)chromone | TERT | O14746 | √ |
| MOL003095 | 5-hydroxy-7-methoxy-2-(3,4,5-trimethoxyphenyl)chromone | GLO1 | Q04760 | √ |
| MOL003095 | 5-hydroxy-7-methoxy-2-(3,4,5-trimethoxyphenyl)chromone | ESR2 | Q92731 | √ |
| MOL003095 | 5-hydroxy-7-methoxy-2-(3,4,5-trimethoxyphenyl)chromone | FLT3 | P36888 | √ |
| MOL003095 | 5-hydroxy-7-methoxy-2-(3,4,5-trimethoxyphenyl)chromone | PIM1 | P11309 | √ |
| MOL003095 | 5-hydroxy-7-methoxy-2-(3,4,5-trimethoxyphenyl)chromone | KIT | P10721 | √ |
| MOL003095 | 5-hydroxy-7-methoxy-2-(3,4,5-trimethoxyphenyl)chromone | GSK3B | P49841 | √ |
| MOL003095 | 5-hydroxy-7-methoxy-2-(3,4,5-trimethoxyphenyl)chromone | HSD17B1 | P14061 | √ |
| MOL003095 | 5-hydroxy-7-methoxy-2-(3,4,5-trimethoxyphenyl)chromone | CSNK2A1 | P68400 | √ |
| MOL003095 | 5-hydroxy-7-methoxy-2-(3,4,5-trimethoxyphenyl)chromone | MAOA | P21397 | √ |
| MOL003095 | 5-hydroxy-7-methoxy-2-(3,4,5-trimethoxyphenyl)chromone | GRK6 | P43250 | √ |
| MOL003095 | 5-hydroxy-7-methoxy-2-(3,4,5-trimethoxyphenyl)chromone | OPRM1 | P35372 | √ |
| MOL003095 | 5-hydroxy-7-methoxy-2-(3,4,5-trimethoxyphenyl)chromone | PTGS2 | P35354 | √ |
| MOL003095 | 5-hydroxy-7-methoxy-2-(3,4,5-trimethoxyphenyl)chromone | ST6GAL1 | P15907 | √ |
| MOL003095 | 5-hydroxy-7-methoxy-2-(3,4,5-trimethoxyphenyl)chromone | MAPT | P10636 | √ |
| MOL003095 | 5-hydroxy-7-methoxy-2-(3,4,5-trimethoxyphenyl)chromone | KDM4E | B2RXH2 | √ |
| MOL003095 | 5-hydroxy-7-methoxy-2-(3,4,5-trimethoxyphenyl)chromone | GPR35 | Q9HC97 | √ |
| MOL003095 | 5-hydroxy-7-methoxy-2-(3,4,5-trimethoxyphenyl)chromone | TOP2A | P11388 | √ |
| MOL003095 | 5-hydroxy-7-methoxy-2-(3,4,5-trimethoxyphenyl)chromone | IGF1R | P08069 | √ |
| MOL003095 | 5-hydroxy-7-methoxy-2-(3,4,5-trimethoxyphenyl)chromone | INSR | P06213 | √ |
| MOL003095 | 5-hydroxy-7-methoxy-2-(3,4,5-trimethoxyphenyl)chromone | F2 | P00734 | √ |
| MOL003095 | 5-hydroxy-7-methoxy-2-(3,4,5-trimethoxyphenyl)chromone | DRD4 | P21917 | √ |
| MOL003095 | 5-hydroxy-7-methoxy-2-(3,4,5-trimethoxyphenyl)chromone | MYLK | Q15746 | √ |
| MOL003095 | 5-hydroxy-7-methoxy-2-(3,4,5-trimethoxyphenyl)chromone | MPO | P05164 | √ |
| MOL003095 | 5-hydroxy-7-methoxy-2-(3,4,5-trimethoxyphenyl)chromone | PIK3R1 | P27986 | √ |
| MOL003095 | 5-hydroxy-7-methoxy-2-(3,4,5-trimethoxyphenyl)chromone | DAPK1 | P53355 | √ |
| MOL003095 | 5-hydroxy-7-methoxy-2-(3,4,5-trimethoxyphenyl)chromone | PYGL | P06737 | √ |
| MOL003095 | 5-hydroxy-7-methoxy-2-(3,4,5-trimethoxyphenyl)chromone | PTK2 | Q05397 | √ |
| MOL003095 | 5-hydroxy-7-methoxy-2-(3,4,5-trimethoxyphenyl)chromone | HSD17B2 | P37059 | √ |
| MOL003095 | 5-hydroxy-7-methoxy-2-(3,4,5-trimethoxyphenyl)chromone | KDR | P35968 | √ |
| MOL003095 | 5-hydroxy-7-methoxy-2-(3,4,5-trimethoxyphenyl)chromone | PLK1 | P53350 | √ |
| MOL003095 | 5-hydroxy-7-methoxy-2-(3,4,5-trimethoxyphenyl)chromone | PKN1 | Q16512 | √ |
| MOL003095 | 5-hydroxy-7-methoxy-2-(3,4,5-trimethoxyphenyl)chromone | MET | P08581 | √ |
| MOL003095 | 5-hydroxy-7-methoxy-2-(3,4,5-trimethoxyphenyl)chromone | NEK2 | P51955 | √ |
| MOL003095 | 5-hydroxy-7-methoxy-2-(3,4,5-trimethoxyphenyl)chromone | NEK6 | Q9HC98 | √ |
| MOL003095 | 5-hydroxy-7-methoxy-2-(3,4,5-trimethoxyphenyl)chromone | PLA2G1B | P04054 | √ |
| MOL003095 | 5-hydroxy-7-methoxy-2-(3,4,5-trimethoxyphenyl)chromone | NUAK1 | O60285 | √ |
| MOL003095 | 5-hydroxy-7-methoxy-2-(3,4,5-trimethoxyphenyl)chromone | CDK2 | P24941 | √ |
| MOL003095 | 5-hydroxy-7-methoxy-2-(3,4,5-trimethoxyphenyl)chromone | MMP13 | P45452 | √ |
| MOL003095 | 5-hydroxy-7-methoxy-2-(3,4,5-trimethoxyphenyl)chromone | MMP3 | P08254 | √ |
| MOL003095 | 5-hydroxy-7-methoxy-2-(3,4,5-trimethoxyphenyl)chromone | SRC | P12931 | √ |
| MOL003095 | 5-hydroxy-7-methoxy-2-(3,4,5-trimethoxyphenyl)chromone | CYP19A1 | P11511 | √ |
| MOL003095 | 5-hydroxy-7-methoxy-2-(3,4,5-trimethoxyphenyl)chromone | TNKS2 | Q9H2K2 | √ |
| MOL003095 | 5-hydroxy-7-methoxy-2-(3,4,5-trimethoxyphenyl)chromone | TNKS | O95271 | √ |
| MOL003095 | 5-hydroxy-7-methoxy-2-(3,4,5-trimethoxyphenyl)chromone | ODC1 | P11926 | √ |
| MOL003095 | 5-hydroxy-7-methoxy-2-(3,4,5-trimethoxyphenyl)chromone | PFKFB3 | Q16875 | √ |
| MOL003095 | 5-hydroxy-7-methoxy-2-(3,4,5-trimethoxyphenyl)chromone | EGFR | P00533 | √ |
| MOL003095 | 5-hydroxy-7-methoxy-2-(3,4,5-trimethoxyphenyl)chromone | CXCR1 | P25024 | √ |
| MOL003095 | 5-hydroxy-7-methoxy-2-(3,4,5-trimethoxyphenyl)chromone | CDK1 | P06493 | √ |
| MOL003095 | 5-hydroxy-7-methoxy-2-(3,4,5-trimethoxyphenyl)chromone | ABCC1 | P33527 | × |
| MOL003095 | 5-hydroxy-7-methoxy-2-(3,4,5-trimethoxyphenyl)chromone | AKR1B1 | P15121 | × |
| MOL003095 | 5-hydroxy-7-methoxy-2-(3,4,5-trimethoxyphenyl)chromone | ADORA2A | P29274 | × |
| MOL003095 | 5-hydroxy-7-methoxy-2-(3,4,5-trimethoxyphenyl)chromone | CA2 | P00918 | × |
| MOL003095 | 5-hydroxy-7-methoxy-2-(3,4,5-trimethoxyphenyl)chromone | CA12 | O43570 | × |
| MOL003095 | 5-hydroxy-7-methoxy-2-(3,4,5-trimethoxyphenyl)chromone | CA7 | P43166 | × |
| MOL003095 | 5-hydroxy-7-methoxy-2-(3,4,5-trimethoxyphenyl)chromone | APP | P05067 | × |
| MOL003095 | 5-hydroxy-7-methoxy-2-(3,4,5-trimethoxyphenyl)chromone | CA4 | P22748 | × |
| MOL003095 | 5-hydroxy-7-methoxy-2-(3,4,5-trimethoxyphenyl)chromone | AKR1B10 | O60218 | × |
| MOL003095 | 5-hydroxy-7-methoxy-2-(3,4,5-trimethoxyphenyl)chromone | ARG1 | P05089 | × |
| MOL003095 | 5-hydroxy-7-methoxy-2-(3,4,5-trimethoxyphenyl)chromone | ADORA3 | P0DMS8 | × |
| MOL003095 | 5-hydroxy-7-methoxy-2-(3,4,5-trimethoxyphenyl)chromone | ABCG2 | Q9UNQ0 | × |
| MOL003095 | 5-hydroxy-7-methoxy-2-(3,4,5-trimethoxyphenyl)chromone | ADORA1 | P30542 | × |
| MOL003095 | 5-hydroxy-7-methoxy-2-(3,4,5-trimethoxyphenyl)chromone | AMY1A | P04745 | × |
| MOL003095 | 5-hydroxy-7-methoxy-2-(3,4,5-trimethoxyphenyl)chromone | ABCB1 | P08183 | × |
| MOL003095 | 5-hydroxy-7-methoxy-2-(3,4,5-trimethoxyphenyl)chromone | ALOX5 | P09917 | × |
| MOL003095 | 5-hydroxy-7-methoxy-2-(3,4,5-trimethoxyphenyl)chromone | ACHE | P22303 | × |
| MOL003095 | 5-hydroxy-7-methoxy-2-(3,4,5-trimethoxyphenyl)chromone | CA1 | P00915 | × |
| MOL003095 | 5-hydroxy-7-methoxy-2-(3,4,5-trimethoxyphenyl)chromone | CA9 | Q16790 | × |
| MOL003095 | 5-hydroxy-7-methoxy-2-(3,4,5-trimethoxyphenyl)chromone | AR | P10275 | × |
| MOL003095 | 5-hydroxy-7-methoxy-2-(3,4,5-trimethoxyphenyl)chromone | CBR1 | P16152 | × |
| MOL003095 | 5-hydroxy-7-methoxy-2-(3,4,5-trimethoxyphenyl)chromone | AURKB | Q96GD4 | × |
| MOL003095 | 5-hydroxy-7-methoxy-2-(3,4,5-trimethoxyphenyl)chromone | CA3 | P07451 | × |
| MOL003095 | 5-hydroxy-7-methoxy-2-(3,4,5-trimethoxyphenyl)chromone | CA6 | P23280 | × |
| MOL003095 | 5-hydroxy-7-methoxy-2-(3,4,5-trimethoxyphenyl)chromone | CAMK2B | Q13554 | × |
| MOL003095 | 5-hydroxy-7-methoxy-2-(3,4,5-trimethoxyphenyl)chromone | ALK | Q9UM73 | × |
| MOL003095 | 5-hydroxy-7-methoxy-2-(3,4,5-trimethoxyphenyl)chromone | CA5A | P35218 | × |
| MOL003095 | 5-hydroxy-7-methoxy-2-(3,4,5-trimethoxyphenyl)chromone | AXL | P30530 | × |
| MOL003095 | 5-hydroxy-7-methoxy-2-(3,4,5-trimethoxyphenyl)chromone | APEX1 | P27695 | × |
| MOL003095 | 5-hydroxy-7-methoxy-2-(3,4,5-trimethoxyphenyl)chromone | AKR1C2 | P52895 | × |
| MOL003095 | 5-hydroxy-7-methoxy-2-(3,4,5-trimethoxyphenyl)chromone | AKR1C1 | Q04828 | × |
| MOL003095 | 5-hydroxy-7-methoxy-2-(3,4,5-trimethoxyphenyl)chromone | AKR1C3 | P42330 | × |
| MOL003095 | 5-hydroxy-7-methoxy-2-(3,4,5-trimethoxyphenyl)chromone | AKR1C4 | P17516 | × |
| MOL003095 | 5-hydroxy-7-methoxy-2-(3,4,5-trimethoxyphenyl)chromone | CA13 | Q8N1Q1 | × |
| MOL003095 | 5-hydroxy-7-methoxy-2-(3,4,5-trimethoxyphenyl)chromone | CA14 | Q9ULX7 | × |
| MOL003095 | 5-hydroxy-7-methoxy-2-(3,4,5-trimethoxyphenyl)chromone | BCL2 | P10415 | × |
| MOL003095 | 5-hydroxy-7-methoxy-2-(3,4,5-trimethoxyphenyl)chromone | CCNB3 | Q8WWL7 | × |
| MOL003095 | 5-hydroxy-7-methoxy-2-(3,4,5-trimethoxyphenyl)chromone | CCNB1 | P14635 | × |
| MOL003095 | 5-hydroxy-7-methoxy-2-(3,4,5-trimethoxyphenyl)chromone | CCNB2 | O95067 | × |
| MOL003101 | 7-epi-Vogeloside | SLC5A2 | P31639 | √ |
| MOL003101 | 7-epi-Vogeloside | LGALS3 | P17931 | √ |
| MOL003101 | 7-epi-Vogeloside | LGALS9 | O00182 | √ |
| MOL003101 | 7-epi-Vogeloside | SLC29A1 | Q99808 | √ |
| MOL003101 | 7-epi-Vogeloside | SLC5A1 | P13866 | √ |
| MOL003101 | 7-epi-Vogeloside | HK2 | P52789 | √ |
| MOL003101 | 7-epi-Vogeloside | HK1 | P19367 | √ |
| MOL003101 | 7-epi-Vogeloside | GBA | P04062 | √ |
| MOL003101 | 7-epi-Vogeloside | EGFR | P00533 | √ |
| MOL003101 | 7-epi-Vogeloside | HSPA8 | P11142 | √ |
| MOL003101 | 7-epi-Vogeloside | HSPA5 | P11021 | √ |
| MOL003101 | 7-epi-Vogeloside | SLC5A4 | Q9NY91 | √ |
| MOL003101 | 7-epi-Vogeloside | TYR | P14679 | √ |
| MOL003101 | 7-epi-Vogeloside | EDNRA | P25101 | √ |
| MOL003101 | 7-epi-Vogeloside | MMP13 | P45452 | √ |
| MOL003101 | 7-epi-Vogeloside | MMP1 | P03956 | √ |
| MOL003101 | 7-epi-Vogeloside | MMP7 | P09237 | √ |
| MOL003101 | 7-epi-Vogeloside | MMP12 | P39900 | √ |
| MOL003101 | 7-epi-Vogeloside | MMP8 | P22894 | √ |
| MOL003101 | 7-epi-Vogeloside | CTSK | P43235 | √ |
| MOL003101 | 7-epi-Vogeloside | CTSS | P25774 | √ |
| MOL003101 | 7-epi-Vogeloside | CTSL | P07711 | √ |
| MOL003101 | 7-epi-Vogeloside | OGA | O60502 | √ |
| MOL003101 | 7-epi-Vogeloside | LGALS7 | P47929 | √ |
| MOL003101 | 7-epi-Vogeloside | FUCA1 | P04066 | √ |
| MOL003101 | 7-epi-Vogeloside | PDE5A | O76074 | √ |
| MOL003101 | 7-epi-Vogeloside | MAPK10 | P53779 | √ |
| MOL003101 | 7-epi-Vogeloside | ADORA1 | P30542 | × |
| MOL003101 | 7-epi-Vogeloside | ADORA2A | P29274 | × |
| MOL003101 | 7-epi-Vogeloside | ADORA3 | P0DMS8 | × |
| MOL003101 | 7-epi-Vogeloside | ADK | P55263 | × |
| MOL003101 | 7-epi-Vogeloside | CA2 | P00918 | × |
| MOL003101 | 7-epi-Vogeloside | CA1 | P00915 | × |
| MOL003101 | 7-epi-Vogeloside | CA12 | O43570 | × |
| MOL003101 | 7-epi-Vogeloside | CA14 | Q9ULX7 | × |
| MOL003101 | 7-epi-Vogeloside | CA9 | Q16790 | × |
| MOL003101 | 7-epi-Vogeloside | ADORA2B | P29275 | × |
| MOL003101 | 7-epi-Vogeloside | AGTR1 | P30556 | × |
| MOL003101 | 7-epi-Vogeloside | AKR1B1 | P15121 | × |
| MOL003108 | Caeruloside C | EPHX2 | P34913 | √ |
| MOL003108 | Caeruloside C | CHIA | Q9BZP6 | √ |
| MOL003108 | Caeruloside C | TYR | P14679 | √ |
| MOL003108 | Caeruloside C | SLC29A1 | Q99808 | √ |
| MOL003108 | Caeruloside C | CA2 | P00918 | × |
| MOL003108 | Caeruloside C | CA1 | P00915 | × |
| MOL003108 | Caeruloside C | CA12 | O43570 | × |
| MOL003108 | Caeruloside C | CA9 | Q16790 | × |
| MOL003108 | Caeruloside C | ADORA1 | P30542 | × |
| MOL003108 | Caeruloside C | ADORA2A | P29274 | × |
| MOL003108 | Caeruloside C | ADORA2B | P29275 | × |
| MOL003108 | Caeruloside C | ADORA3 | P0DMS8 | × |
| MOL003117 | Ioniceracetalides B_qt | MMP2 | P08253 | √ |
| MOL003117 | Ioniceracetalides B_qt | HDAC6 | Q9UBN7 | √ |
| MOL003117 | Ioniceracetalides B_qt | HDAC8 | Q9BY41 | √ |
| MOL003117 | Ioniceracetalides B_qt | HDAC1 | Q13547 | √ |
| MOL003117 | Ioniceracetalides B_qt | MMP13 | P45452 | √ |
| MOL003117 | Ioniceracetalides B_qt | MMP3 | P08254 | √ |
| MOL003117 | Ioniceracetalides B_qt | MMP1 | P03956 | √ |
| MOL003117 | Ioniceracetalides B_qt | CA2 | P00918 | × |
| MOL003117 | Ioniceracetalides B_qt | AKT1 | P31749 | × |
| MOL003128 | dinethylsecologanoside | SLC29A1 | Q99808 | √ |
| MOL003128 | dinethylsecologanoside | HK2 | P52789 | √ |
| MOL003128 | dinethylsecologanoside | HK1 | P19367 | √ |
| MOL003128 | dinethylsecologanoside | LGALS3 | P17931 | √ |
| MOL003128 | dinethylsecologanoside | LGALS9 | O00182 | √ |
| MOL003128 | dinethylsecologanoside | SLC5A4 | Q9NY91 | √ |
| MOL003128 | dinethylsecologanoside | SLC5A2 | P31639 | √ |
| MOL003128 | dinethylsecologanoside | SLC5A1 | P13866 | √ |
| MOL003128 | dinethylsecologanoside | MAPK14 | Q16539 | √ |
| MOL003128 | dinethylsecologanoside | EGFR | P00533 | √ |
| MOL003128 | dinethylsecologanoside | LGALS7 | P47929 | √ |
| MOL003128 | dinethylsecologanoside | SLC28A2 | O43868 | √ |
| MOL003128 | dinethylsecologanoside | MAP2K1 | Q02750 | √ |
| MOL003128 | dinethylsecologanoside | HSPA8 | P11142 | √ |
| MOL003128 | dinethylsecologanoside | HSPA5 | P11021 | √ |
| MOL003128 | dinethylsecologanoside | SLC2A1 | P11166 | √ |
| MOL003128 | dinethylsecologanoside | GBA | P04062 | √ |
| MOL003128 | dinethylsecologanoside | MMP3 | P08254 | √ |
| MOL003128 | dinethylsecologanoside | MMP9 | P14780 | √ |
| MOL003128 | dinethylsecologanoside | MMP1 | P03956 | √ |
| MOL003128 | dinethylsecologanoside | PYGM | P11217 | √ |
| MOL003128 | dinethylsecologanoside | ADORA1 | P30542 | × |
| MOL003128 | dinethylsecologanoside | ADORA2A | P29274 | × |
| MOL003128 | dinethylsecologanoside | ADORA3 | P0DMS8 | × |
| MOL003128 | dinethylsecologanoside | AKR1B1 | P15121 | × |
| MOL003128 | dinethylsecologanoside | CA2 | P00918 | × |
| MOL003128 | dinethylsecologanoside | ADORA2B | P29275 | × |
| MOL003128 | dinethylsecologanoside | CA1 | P00915 | × |
| MOL003128 | dinethylsecologanoside | CA12 | O43570 | × |
| MOL003128 | dinethylsecologanoside | CA14 | Q9ULX7 | × |
| MOL003128 | dinethylsecologanoside | CA9 | Q16790 | × |
| MOL003128 | dinethylsecologanoside | ADK | P55263 | × |
| MOL003128 | dinethylsecologanoside | ADAM17 | P78536 | × |
