# Supplementary material for: Network pharmacology combined with metabolomics to explore the mechanism for Lonicerae Japonicae flos against respiratory syncytial virus
Source: BMC Complement Med Ther. 2023 Dec 12;23:449. doi: 10.1186/s12906-023-04286-0 (PMC10714634; doi:10.1186/s12906-023-04286-0)
Supplement: Supplementary file 3 — Additional file 3: Supplementary Table S3. RSV infection-related genes. [file 12906_2023_4286_MOESM3_ESM.docx]

**Network pharmacology combined with metabolomics to explore the mechanism for *Lonicerae japonicae* flos against Respiratory Syncytial Virus**

Jie Ding^1^, Jing Li^1^, Zhe Zhang^1^, Yaxuan Du^2^, Yuhong Liu^1, *^, Ping Wang^3, *^, Haitao Du^3, *^

^1^ College of Pharmacy, Shandong University of Traditional Chinese Medicine, Jinan, 250355, China

^2^ School of Chinese Materia Medica, Shenyang Pharmaceutical University, Shenyang 117004, China

^3^ Shandong Academy of Chinese Medicine, Jinan, 250014, China

^*^Corresponding author. Yuhong Liu, Shandong University of Traditional Chinese Medicine, Jinan, 250355, China. Ping Wang and Haitao Du, Shandong Academy of Chinese Medicine, Jinan, 250014, China.

E-mail addresses: liuyuhongwu@126.com (Yuhong Liu), wangpingjinan@126.com (Ping Wang), kkitdht@foxmail.com (Haitao Du).

**Supplementary Table S3. RSV infection-related genes**

| Gene Symbol |
| --- |
| IL10 |
| NFKB1 |
| RELA |
| ICAM1 |
| AKT1 |
| IL6 |
| TNF |
| TLR3 |
| MTOR |
| TLR4 |
| IL4 |
| TGFB1 |
| IFNG |
| NFKBIA |
| CXCL8 |
| CCL5 |
| IFNA1 |
| FCGR3A |
| TNFSF10 |
| MYC |
| PRF1 |
| GZMB |
| MUC1 |
| IL7R |
| PTPN11 |
| CD38 |
| JAK1 |
| NLRP3 |
| EIF2S1 |
| TLR2 |
| CXCR4 |
| TNFRSF10B |
| IL12B |
| MCL1 |
| MAPK14 |
| SPP1 |
| STAT3 |
| MAPK8 |
| MMP9 |
| JUN |
| NFE2L2 |
| CCL2 |
| TP53 |
| IL1B |
| MAPK1 |
| OAS1 |
| STAT1 |
| CCR5 |
| EGFR |
| IL17A |
| MAVS |
| SFTPD |
| CD14 |
| RSAD2 |
| TLR7 |
| CX3CR1 |
| CCR7 |
| IFNAR1 |
| CCR2 |
| FCGR1A |
| FCGR2A |
| EPO |
| EIF4EBP1 |
| IL2 |
| CASP8 |
| VEGFA |
| CCND1 |
| CDKN1A |
| CASP3 |
| DNAAF5 |
| KLRK1 |
| HTRA2 |
| ITGAL |
| TRAF6 |
| AKT2 |
| IRAK1 |
| ITGAV |
| KPNA2 |
| ABL1 |
| SPHK1 |
| NFKB2 |
| CSF3 |
| KIT |
| LMNA |
| IL1R1 |
| CHUK |
| IL12A |
| MYD88 |
| ERN1 |
| THRA |
| CYBA |
| SOCS3 |
| IL5 |
| IKBKB |
| ERBB2 |
| PRKCD |
| ATM |
| RAF1 |
| HRAS |
| FASLG |
| G6PD |
| CCL3 |
| MDM2 |
| MAPK9 |
| CDK1 |
| CXCL1 |
| SERPINE1 |
| HIF1A |
| BCL2L1 |
| ABCB1 |
| PARP1 |
| BAX |
| PTGS2 |
| BCL2 |
| MAPK3 |
| IFNA2 |
| IL13 |
| DDX58 |
| IFIH1 |
| IRF3 |
| HLA-B |
| CD8A |
| CD4 |
| IFNB1 |
| MBL2 |
| CR2 |
| IL4R |
| STAT2 |
| TFRC |
| PML |
| EIF2AK2 |
| NOS2 |
| CD55 |
| IVNS1ABP |
| MX1 |
| ISG15 |
| IL1A |
| TLR9 |
| IL18 |
| SFTPC |
| CD46 |
| CLEC4M |
| PDCD1 |
| HAVCR2 |
| ITGAM |
| NCF2 |
| CASP1 |
| IL15 |
| HAVCR1 |
| NGF |
| LEP |
| LTF |
| SRC |
| FOS |
| APOE |
| TSLP |
| IL21 |
| C1QBP |
| CD59 |
| SLC2A1 |
| SFTPA1 |
| ABCA3 |
| MUC5AC |
| HMGB1 |
| ETS1 |
| ADRB2 |
| SCGB1A1 |
| SP1 |
| IL11 |
| VDR |
| CCL17 |
| IL33 |
| SMPD1 |
| TLR8 |
| SFTPA2 |
| ERCC2 |
| RELB |
| IKBKE |
| SOCS1 |
| NCL |
| CX3CL1 |
| F9 |
| HPD |
| F |
| FCGR3B |
| C1R |
| C1QA |
| C1QB |
| C1QC |
| FCGR2B |
| FCGR2C |
| SKP2 |
| CRYAB |
| CASP7 |
| EGR1 |
| FAS |
| NQO1 |
| SOD2 |
| KRI1 |
| ELP5 |
| ZNRF2 |
| RDH13 |
| BIRC7 |
| EDARADD |
| SNRNP48 |
| PPP2R5D |
| SPSB3 |
| KATNAL1 |
| TMEM263 |
| GALNT4 |
| MRPS12 |
| SLC26A11 |
| PRPF6 |
| LST1 |
| CTDSPL2 |
| HNRNPLL |
| TMEM41B |
| ATE1 |
| ERLIN2 |
| OGFR |
| TSEN15 |
| MARK2 |
| AP2A1 |
| BRCC3 |
| DCPS |
| LSM4 |
| NAIP |
| SLC25A11 |
| XPO5 |
| PHTF1 |
| MRPL19 |
| NLRP12 |
| SRRT |
| NADK2 |
| SMC6 |
| SALL4 |
| AGTRAP |
| ERCC3 |
| SSRP1 |
| ABI1 |
| IFRD2 |
| CYB5R1 |
| SUV39H1 |
| SMC3 |
| UQCRB |
| COX8A |
| MBD1 |
| DNM2 |
| RB1CC1 |
| RECQL4 |
| BCR |
| ADAM10 |
| RAPH1 |
| ASAP1 |
| GSPT1 |
| TRA2A |
| PRDM2 |
| CDC34 |
| TCF7 |
| CLEC7A |
| EPHA1 |
| RAB8B |
| RPA3 |
| SCPEP1 |
| RDH11 |
| ALOX12 |
| GSK3A |
| PPM1A |
| SUMO1 |
| RIPK3 |
| YPEL5 |
| CHAF1A |
| CCL8 |
| DBN1 |
| GLA |
| ACOT7 |
| AFF4 |
| IDE |
| CBL |
| FAM43A |
| MYO5A |
| VEGFB |
| CRIP2 |
| ETV4 |
| PSMD12 |
| BCL2L2 |
| MFN1 |
| TFDP1 |
| LIMA1 |
| LRRFIP1 |
| RPS19 |
| SMC1A |
| TXN2 |
| ALDH18A1 |
| EIF1 |
| SRSF1 |
| FOXA1 |
| TOP2B |
| FZD2 |
| BARD1 |
| FMR1 |
| PSMB5 |
| RIPK1 |
| NABP1 |
| TCF7L2 |
| AHCY |
| CD74 |
| IRAK2 |
| KAT2B |
| RAC2 |
| TCF19 |
| ARHGDIA |
| FANCD2 |
| RRM2B |
| FBLN5 |
| YWHAE |
| CD69 |
| NOTCH2 |
| SAA1 |
| EIF5A |
| NCOA3 |
| CDT1 |
| OGDH |
| YWHAZ |
| EIF4E |
| NQO2 |
| TAGLN2 |
| BIK |
| CBR3 |
| ESRRA |
| MSH6 |
| SHC1 |
| IGFBP2 |
| VASP |
| IDH3A |
| IFITM1 |
| THY1 |
| TNFRSF9 |
| DLGAP5 |
| S100A4 |
| TMPO |
| HSPA4L |
| FGF21 |
| TCF4 |
| SIGMAR1 |
| CDC42 |
| SLC31A1 |
| CCNF |
| MCM7 |
| BCL2A1 |
| PTX3 |
| NFKBIB |
| SPTBN1 |
| STIP1 |
| UPP1 |
| BCL3 |
| WEE1 |
| BRCA2 |
| HSF1 |
| DIABLO |
| ATG5 |
| MAP2K6 |
| TNFRSF10A |
| GHR |
| TPI1 |
| CREM |
| ENO2 |
| B2M |
| BCL6 |
| ATF2 |
| VDAC1 |
| TGFB2 |
| MAP2K2 |
| AQP3 |
| NR1H3 |
| JUND |
| CREBBP |
| SLC7A5 |
| CASP2 |
| TXN |
| CASP6 |
| GADD45G |
| CASP4 |
| PDGFRB |
| FKBP5 |
| BIRC2 |
| CASP12 |
| HSPA1B |
| PPARD |
| ADM |
| RAD51 |
| BRCA1 |
| IGF2 |
| NUPR1 |
| RPS6 |
| AKR1B1 |
| AKR1B10 |
| CFLAR |
| E2F1 |
| HSP90AB1 |
| TNFAIP3 |
| EIF2AK3 |
| BBC3 |
| XDH |
| ME1 |
| SCD |
| GADD45B |
| NDRG1 |
| IRS1 |
| MGMT |
| BAK1 |
| SNCA |
| PMAIP1 |
| ACE |
| AIFM1 |
| RUNX2 |
| BCL2L11 |
| CCND2 |
| CTSD |
| BECN1 |
| HSP90AA1 |
| AKR1C3 |
| IGF1R |
| KDR |
| MAP2K1 |
| ABCC4 |
| BIRC3 |
| CEBPA |
| ATF4 |
| CLU |
| XBP1 |
| F3 |
| RPS6KB1 |
| XIAP |
| GDF15 |
| HSPB1 |
| CYP2C19 |
| NR1H4 |
| GPT |
| HSPA1A |
| MKI67 |
| CEBPB |
| BID |
| CYP2D6 |
| CDK2 |
| ABCC1 |
| CYP2C9 |
| PPARGC1A |
| SQSTM1 |
| CXCL2 |
| ATF3 |
| PGR |
| CDKN1B |
| RB1 |
| GADD45A |
| CYCS |
| BIRC5 |
| CCNB1 |
| GSK3B |
| CTNNB1 |
| ABCC2 |
| ESR2 |
| NR1I3 |
| HSPA5 |
| DDIT3 |
| GSR |
| AR |
| ESR1 |
| CYP3A4 |
| CYP1A2 |
| CASP9 |
| HMOX1 |
| SYNJ2BP-COX16 |
| CD40LG |
| CD209 |
| IRF1 |
| SFTPB |
| CCL11 |
| HLA-A |
| ELANE |
| IL2RA |
| CRP |
| ALB |
| CXCL10 |
| CD79A |
| IRF7 |
| CTLA4 |
| ADA |
| CD40 |
| CR1 |
| IFITM3 |
| FOXP3 |
| TAP2 |
| TNFRSF1A |
| NEU1 |
| TBK1 |
| CXCL12 |
| IFNGR1 |
| CD3D |
| RNASE3 |
| TAP1 |
| IL7 |
| CSF2 |
| HLA-C |
| IL1RN |
| EP300 |
| CCL4 |
| CCR1 |
| NKX2-1 |
| CDH1 |
| CD28 |
| C3 |
| IL2RB |
| SOD1 |
| HP |
| BST2 |
| APOBEC3G |
| CYBB |
| IRAK4 |
| PIK3CD |
| LACTB |
| ATP6AP1 |
| IL12RB1 |
| IFNL3 |
| LTA |
| CCR3 |
| CXCR3 |
| MPO |
| TNFRSF1B |
| NBN |
| ITIH4 |
| IFNAR2 |
| CD19 |
| IL10RB |
| CREB1 |
| CD36 |
| ITGB2 |
| NOD2 |
| PPARG |
| TYK2 |
| BDNF |
| IGF2R |
| ERVW-1 |
| ALOX5 |
| IL3 |
| JAK2 |
| CD274 |
| DEFB4A |
| IL2RG |
| GAA |
| VCAM1 |
| EDN1 |
| THBD |
| LDLR |
| IRF5 |
| C5 |
| CD27 |
| SELP |
| IDO1 |
| TLR5 |
| CXCL9 |
| IL17RA |
| RNASEL |
| NR3C1 |
| TP63 |
| CSF3R |
| CD80 |
| SELL |
| STAT4 |
| PPIA |
| SCNN1B |
| ALK |
| FURIN |
| TICAM1 |
| STAT5B |
| CD86 |
| SELE |
| TKT |
| IL16 |
| CD244 |
| SMAD3 |
| MMP2 |
| KRT18 |
| ITGB1 |
| CXCR2 |
| ACTC1 |
| CD44 |
| GSTM1 |
| TGFBR1 |
| HBB |
| IL10RA |
| DSP |
| TBX21 |
| TF |
| IL6R |
| CAV1 |
| GLI2 |
| LPO |
| IGF1 |
| HSPA4 |
| CDK9 |
| TAC1 |
| FGF2 |
| ANXA5 |
| CALCA |
| CCL22 |
| STAT5A |
| PSMB8 |
| TLR1 |
| PF4 |
| NPM1 |
| SLAMF1 |
| HSPD1 |
| MX2 |
| TLR6 |
| MME |
| CSF1 |
| RHOA |
| NOTCH1 |
| KRT8 |
| MUC5B |
| FCER2 |
| CFD |
| SAMD9 |
| TBX4 |
| MYCL |
| FES |
| SLC1A5 |
| MPL |
| SKI |
| MYCN |
| FGF3 |
| SPI1 |
| MYB |
| MOS |
| KRAS |
| FLI1 |
| ERAS |
| MRAS |
| NDUFS1 |
| NDUFV1 |
| NDUFV2 |
| GABPA |
| NRF1 |
| NDUFS7 |
| NDUFS8 |
| NDUFV3 |
| NDUFS4 |
| NDUFS2 |
| NDUFB6 |
| NDUFS3 |
| NDUFS5 |
| NDUFS6 |
| TLX3 |
| COA7 |
| FLT1 |
| RNASE7 |
| SOX4 |
| LAMTOR5 |
| HIVEP3 |
| NR2F6 |
| NR2F1 |
| SLC20A1 |
| HIVEP2 |
| PSMD7 |
| SLC20A2 |
| EVI2A |
| EVI2B |
| MYBL1 |
| ABL2 |
| ETS2 |
| BRAF |
| CRK |
| CSF1R |
| FOSB |
| NRAS |
| WNT1 |
| BMI1 |
| YES1 |
| REL |
| FGR |
| NOTCH4 |
| ROS1 |
| ERG |
| RRAS |
| LYN |
| MECOM |
| WNT3 |
| RALA |
| RALB |
| RARB |
| PDGFB |
| ERBB3 |
| THRB |
| HIVEP1 |
| THPO |
| RRAS2 |
| DUSP3 |
| ERBB4 |
| MYBL2 |
| TRAF1 |
| CRKL |
| DUSP8 |
| VRK1 |
| VRK2 |
| EIF3E |
| EVI5 |
| PLXNC1 |
| TAX1BP1 |
| AIP |
| GPR183 |
| EBI3 |
| FLVCR1 |
| URGCP |
| MOV10 |
| FLVCR2 |
| RBCK1 |
| AKT3 |
| PDCL3 |
| KIAA0319L |
| ACY3 |
| FAM89B |
| EXOC3L2 |
| DPP4 |
| CD81 |
| SH2D1A |
| SERPINA1 |
| EOMES |
| DEFA1 |
| DMD |
| RPGR |
| IL22 |
| HLA-DRB1 |
| TFAM |
| UBD |
| BTK |
| KLF2 |
| NDUFA13 |
| CD3G |
| STK4 |
| MECP2 |
| ACE2 |
| MICA |
| AGER |
| PRKCA |
| OPRM1 |
| TRPV1 |
| GATA3 |
| NTRK1 |
| IFNL1 |
| BRD4 |
| MICB |
| KLF6 |
| LY96 |
| DUSP1 |
| LIF |
| HMGCR |
| PRDX1 |
| ARG1 |
| EEF1A1 |
| IL9 |
| TRPA1 |
| OGT |
| EFNB2 |
| MAPKAPK2 |
| OPRD1 |
| MMP10 |
| TRIM25 |
| SIGLEC1 |
| IL19 |
| MMP19 |
| ACTR2 |
| CCL7 |
| GZMA |
| VPS4A |
| TNFRSF10D |
| IFI27 |
| ASIC3 |
| DCP1A |
| IFI6 |
