# Supplementary material for: Network pharmacology combined with metabolomics to explore the mechanism for Lonicerae Japonicae flos against respiratory syncytial virus
Source: BMC Complement Med Ther. 2023 Dec 12;23:449. doi: 10.1186/s12906-023-04286-0 (PMC10714634; doi:10.1186/s12906-023-04286-0)
Supplement: Supplementary file 4 — Additional file 4: Supplementary Table S4. Potential target of LJF against RSV infection. [file 12906_2023_4286_MOESM4_ESM.docx]

**Network pharmacology combined with metabolomics to explore the mechanism for *Lonicerae japonicae* flos against Respiratory Syncytial Virus**

Jie Ding^1^, Jing Li^1^, Zhe Zhang^1^, Yaxuan Du^2^, Yuhong Liu^1, *^, Ping Wang^3, *^, Haitao Du^3, *^

^1^ College of Pharmacy, Shandong University of Traditional Chinese Medicine, Jinan, 250355, China

^2^ School of Chinese Materia Medica, Shenyang Pharmaceutical University, Shenyang 117004, China

^3^ Shandong Academy of Chinese Medicine, Jinan, 250014, China

^*^Corresponding author. Yuhong Liu, Shandong University of Traditional Chinese Medicine, Jinan, 250355, China. Ping Wang and Haitao Du, Shandong Academy of Chinese Medicine, Jinan, 250014, China.

E-mail addresses: liuyuhongwu@126.com (Yuhong Liu), wangpingjinan@126.com (Ping Wang), kkitdht@foxmail.com (Haitao Du).

**Supplementary Table S4. Potential target of LJF against RSV infection**

| Gene Symbol |
| --- |
| AKR1B1 |
| XDH |
| CDK1 |
| CCNB1 |
| ALOX5 |
| GSK3B |
| PARP1 |
| MMP9 |
| MMP2 |
| CD38 |
| AKR1B10 |
| ARG1 |
| ABCC1 |
| ABCB1 |
| ESR2 |
| ALOX12 |
| ESR1 |
| PTGS2 |
| CDK2 |
| ESRRA |
| IGF1R |
| EGFR |
| MPO |
| SRC |
| KDR |
| ALK |
| AKT1 |
| AKR1C3 |
| AR |
| NR1H3 |
| HMGCR |
| CYP2C19 |
| G6PD |
| NR1I3 |
| VDR |
| PPARD |
| NOS2 |
| PPARG |
| NR3C1 |
| SIGMAR1 |
| IDO1 |
| MDM2 |
| MAPK3 |
| PTPN11 |
| CXCR3 |
| TNF |
| HIF1A |
| MAPK8 |
| CCR1 |
| ITGAL |
| ICAM1 |
| ITGB2 |
| PGR |
| PRKCD |
| RARB |
| PRKCA |
| FLT1 |
| PDGFRB |
| KIT |
| SCD |
| JAK1 |
| ABL1 |
| JAK2 |
| MAPK1 |
| BRD4 |
| YES1 |
| RAF1 |
| NLRP3 |
| MAPK14 |
| CDK9 |
| BTK |
| SNCA |
| STAT1 |
| SERPINE1 |
| MME |
| ACE |
| SELL |
| SELP |
| CASP3 |
| CASP6 |
| CASP7 |
| CASP8 |
| CASP1 |
| CASP2 |
| CFD |
| STAT3 |
| IRAK4 |
| ERBB2 |
| MTOR |
| CCR3 |
| ADAM10 |
| TYK2 |
| CSF1R |
| CXCR2 |
| CCND1 |
| OPRD1 |
| MCL1 |
| OPRM1 |
| BCL2 |
| HSPA5 |
| MAP2K1 |
| SLC2A1 |
